# Chemical compound cinobufotalin potently induces FOXO1-stimulated cisplatin sensitivity by antagonizing its binding partner MYH9

**DOI:** 10.1038/s41392-019-0084-3

**Published:** 2019-11-18

**Authors:** YongHao Li, Xiong Liu, Xian Lin, Menyang Zhao, Yanyi Xiao, Chen Liu, Zixi Liang, Zelong Lin, Renhui Yi, Zibo Tang, Jiahao Liu, Xin Li, Qingping Jiang, Libo Li, Yinyin Xie, Zhen Liu, Weiyi Fang

**Affiliations:** 1 Cancer Center, Integrated Hospital of Traditional Chinese Medicine, Southern Medical University, 510315 Guangzhou, China; 2 Department of Otolaryngology-Head and Neck Surgery, Nanfang Hospital, Southern Medical University, Guangzhou, China; 3 Cancer Institute, Southern Medical University, 510515 Guangzhou, China; 4 Department of Pathology, Third Affiliated Hospital, Guangzhou Medical University, 510150 Guangzhou, China; 5 Key Laboratory of Protein Modification and Degradation, School of Basic Medical Sciences, Affiliated Cancer Hospital and Institute of Guangzhou Medical University, 511436 Guangzhou, China

**Keywords:** Cancer stem cells, Head and neck cancer, Cancer genomics

## Abstract

In this study, we present novel molecular mechanisms by which FOXO1 functions as a tumor suppressor to prevent the pathogenesis of nasopharyngeal carcinoma (NPC). First, we observed that FOXO1 not only controlled tumor stemness and metastasis, but also sensitized NPC cells to cisplatin (DDP) in vitro and in vivo. Mechanistic studies demonstrated that FOXO1-induced miR-200b expression through the GSK3β/β-catenin/TCF4 network-mediated stimulation of ZEB1, which reduced tumor stemness and the epithelial–mesenchymal transition (EMT) signal. Furthermore, we observed FOXO1 interaction with MYH9 and suppression of MYH9 expression by modulating the PI3K/AKT/c-Myc/P53/miR-133a-3p pathway. Decreased MYH9 expression not only reduced its interactions with GSK3β, but also attenuated TRAF6 expression, which then decreased the ubiquitin-mediated degradation of GSK3β protein. Increased GSK3β expression stimulated the β-catenin/TCF4/ZEB1/miR-200b network, which increased the downstream tumor stemness and EMT signals. Subsequently, we observed that chemically synthesized cinobufotalin (CB) strongly increased FOXO1-induced DDP chemosensitivity by reducing MYH9 expression, and the reduction in MYH9 modulated GSK3β/β-catenin and its downstream tumor stemness and EMT signal in NPC. In clinical samples, the combination of low FOXO1 expression and high MYH9 expression indicated the worst overall survival rates. Our studies demonstrated that CB potently induced FOXO1-mediated DDP sensitivity by antagonizing its binding partner MYH9 to modulate tumor stemness in NPC.

## Introduction

The cancer stem cell (CSC) theory states that tumors are composed of a small group of stem cell-like cells with infinite self-renewal and heterogeneous differentiation abilities and nearby cell clusters that are unevenly differentiated.^1,2^ Abnormal expression of epithelial–mesenchymal transition (EMT)-related genes often leads to tumor cells that have acquired the biological properties of migration and invasion, which eventually induces distant tumor metastases.^3,4^ Numerous studies have clarified that EMT capability is a stem cell characteristic of tumor cells.^5,6^ The presence of CSCs and EMT leads to significant increases in the degree of malignancy associated with tumor cells and further induces tumor cell resistance to radiotherapy and chemotherapy, ultimately reducing the efficacy of tumor treatments.^5,7^ Therefore, exploring stemness mechanisms in tumors and the abnormal expression of EMT-related genes in tumor cells will help to increase our understanding of tumor pathogeneses and improve the design of more effective cancer therapies.

FOXO1, a member of the FOXO family, has been reported in several cancers to be an important tumor suppressor involved in migration, invasion, and metastasis through different mechanisms.^8^ The role of FOXO1 in cancer stemness depends on the tumor type. Cancer stem-like cells in pancreatic ductal adenocarcinoma and gastric cancer cells are FOXO1-negative.^9,10^ In contrast, FOXO1 induces tumor stemness in glioblastoma.^11^ In previous studies, we reported that FOXO1 induces miR-3188 expression, which suppresses nasopharyngeal carcinoma(NPC) growth and 5-Fu chemoresistance.^12^ However, the role of FOXO1 in modulating NPC CSCs, metastatic ability, and cisplatin (DDP) resistance is still unclear.

There is a high incidence of NPC in southern China. NPC is a special type of head and neck cancer characterized by early distant metastases. In clinical medicine, the main cause of death in patients with NPC is closely related to tumor cell metastasis and radio-chemotherapy resistance.^13^

MYH9 encodes a nonmuscle myosin heavy chain IIA protein and is involved in many biological activities that are essential for CSC and EMT, such as cell polarity formation, contraction, migration, and the formation of focal adhesions.^14^ The strong expression of MYH9 induces the development of malignancy characteristics such as tumor invasion and metastatic ability.^15–17^ However, an opposing study demonstrated that MYH9 acts as a tumor suppression factor in head and neck cancers.^18^ Although nasopharyngeal carcinoma belongs in the category of head and neck cancer, the detailed action and molecular mechanism of MYH9 are still unclear in NPC.

Cinobufotalin (CB) is a bufadienolide found in toad venom. Several studies have determined that CB has anticancer activity.^19,20^ However, preclinical evaluation of chemically synthesized CB has rarely been reported. Furthermore, the combined application of CB and DDP has never been reported in tumors.

In this study, we performed a detailed investigation regarding the action of FOXO1 in the pathogenesis of NPC and assessed the role and mechanism of chemically synthesized CB in treating FOXO1-overexpressing NPC cells. The data showed that CB is a potent anti-tumor agent, and it further increased FOXO1-induced DDP chemosensitivity by antagonizing MYH9, which regulates GSK3β/β-catenin-mediated tumor stemness and EMT signals in NPC.

## Results

### FOXO1 inhibits tumor stemness, metastasis, and DDP chemoresistance in NPC

**Fig. 1:**
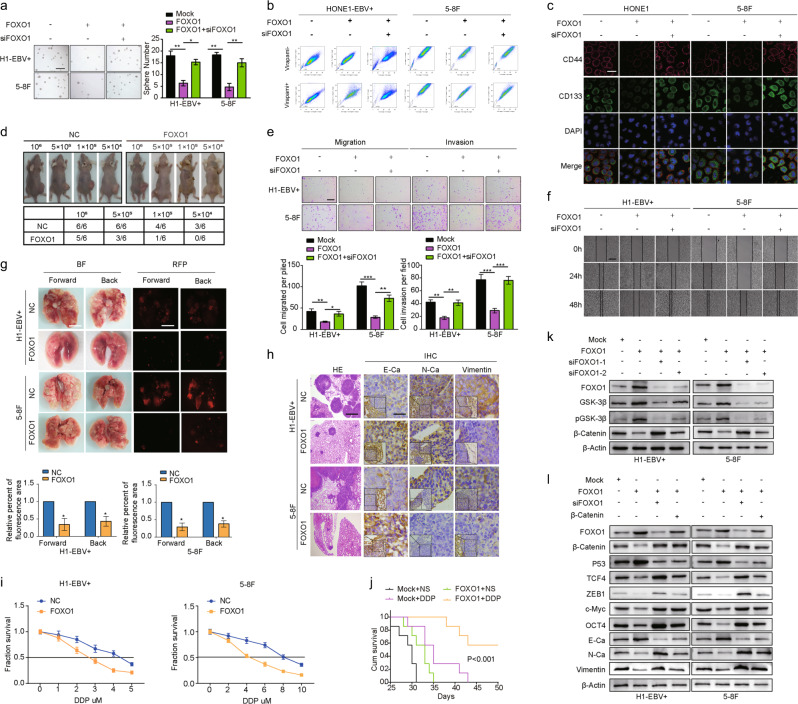
FOXO1 attenuates the stemness, migration, invasion and DDP chemoresistance of NPC cells in vitro and in vivo. **a** Sphere-forming assay (scale bar: 500 μm.), side population assay (**b**), immunofluorescence discrimination assay (**c**, scale bar: 25 μm**)**, migration assay, invasion assays (**e**, scale bar: 200 μm) and scratch assays (**f**, scale bar: 200 μm) of NPC cells were performed after transfection with NC, NC, and/or FOXO1 lentiviral vector, or siRNA as indicated. Student’s *t*-test. Mean ± s.d. **P < 0.01; ***P < 0.001. **d** The in vivo effect of FOXO1 was evaluated in xenograft mouse models bearing tumors originating from HONE1-EBV + and 5–8F cells, *n* = 6/group. **g** Pulmonary metastases were assessed following mouse tail vein injections of HONE1-EBV + and 5–8F cells, *n* = 6/group. **h** Representative H&E staining and IHC staining for E-Ca, N-Ca, and Vimentin in primary tumor tissues are shown. Scale bar: 60 mm. **i** Dose–response curves of HONE1-EBV + and 5–8F cells treated with NC or FOXO1 48 h after treatment with DDP. A parametric generalized linear model with random effects is shown. **j** Survival analysis showed cumulative overall survival time ranked low to high, as follows: Mock + NS < Mock + DDP < FOXO1 + NS < FOXO1 + DDP, *n* = 5/group. Log-rank tests were used for analysis. **k** FOXO1, GSK3β, p-GSK3β, and β-catenin were measured by western blot after transfection with mock and FOXO1 vectors, siFOXO1–1, and siFOXO1–2 as indicated. β-Actin served as a loading control. **l** FOXO1, β-catenin, P53, TCF4, ZEB1, c-Myc, OCT4, E-Ca, N-Ca, and Vimentin were measured by western blot after transfection with mock and FOXO1 vectors, siFOXO1, and β-catenin as indicated. β-Actin served as a loading control

We used a lentiviral vector to overexpress FOXO1 in the NPC cell lines HONE1-EBV + and 5–8F (Supplementary Fig. 1A). Significant FOXO1 upregulation was confirmed for each cell line (Supplementary Fig. 1B). The size and number of tumorspheres reflected the self-renewal ability of CSCs. FOXO1-overexpressing cells formed smaller and less abundant tumorspheres than the vector control cells did in both NPC cell lines (Fig. 1a). Side population (SP) analysis showed that both NPC cell lines overexpressing FOXO1 had a dramatically decreased percentage of SP cells compared with the control groups (from 1.22% to 0.246% and from 3.11% to 0.817%, Fig. 1b). We also observed that FOXO1-overexpressing NPC cells exhibited lower expression of CSC markers (CD133 and CD44) compared with that of the vector control cells, as shown by an immunofluorescence discrimination assay (Fig. 1c). These effects were reversed by transfecting siRNA targeting FOXO1 into FOXO1-overexpressing NPC cells (Supplementary Fig. 1B and Fig. 1a–c). To assess the CSC characteristics of FOXO1 in vivo, BALB/C nude mice were injected with FOXO1-overexpressing 5–8F and HONE1-EBV + cells. As shown in Fig. 1d, when 1 × 10^6^, 5 × 10^5^, 1 × 10^5^, and 5 × 10^4^ LV-FOXO1-RFP 5–8F cells were injected, the rate of tumorigenicity was 83.4% (5/6), 50% (3/6), 16.7% (1/6), and 0% (0/6), respectively, while the rate of tumorigenicity was 100% (6/6), 100% (6/6), 66.7% (4/6), and 50% (3/6), respectively, when cells were injected from the control group. Therefore, these data indicated that FOXO1 inhibits cell stemness in NPC cell lines.

Subsequently, cell migration and invasion were reduced in the FOXO1-overexpressing group compared with that observed in the control cells, while cell migration and invasion were induced in the si-FOXO1 group compared with that observed in the control cells (Fig. 1e, f). To further measure NPC metastasis in vivo, we generated a lung metastasis model by introducing FOXO1-overexpressing and control cells into nude mice through tail vein injections. Six weeks after the injections, the mice injected with HONE1-EBV + -FOXO1 and 5–8F-FOXO1 cells had smaller and fewer pulmonary metastases (Fig. 1g) and displayed lower expression of N-Cadherin (N-Ca) and Vimentin and higher expression of E-Cadherin (E-Ca) in pulmonary metastatic tissues relative to controls (Fig. 1h). These results suggested that FOXO1 significantly inhibited NPC migration, invasion, and metastasis in vitro and in vivo.

DDP has been widely used for the treatment of various solid tumors, including NPC. Thus, we examined how ectopic FOXO1 affected inhibition rates. The IC50 of DDP was significantly reduced after FOXO1 overexpression (Fig. 1i). This effect was also observed in vivo with FOXO1-overexpressing 5–8F xenografts. Survival times calculated by Kaplan–Meier analyses revealed that DDP treatment (NC + DDP) or FOXO1 overexpression (FOXO1 + NS) alone prolonged survival compared with untreated normal controls (NC + NS). However, FOXO1 overexpression coupled with DDP treatment (FOXO1 + DDP) significantly improved the survival compared to what was observed in the other three groups (Fig. 1j). Notably, there was no significant difference between the NC + DDP and FOXO1 + NS groups. To determine the mechanisms by which FOXO1 inhibited NPC cell stemness, metastasis, and DDP chemoresistance, the key regulators of cell stemness and EMT were analyzed using western blots. We observed that the introduction of FOXO1 into NPC cells downregulated the levels of N-Ca, Vimentin, OCT4, and SOX2 and upregulated E-Ca (Fig. 1l). Interestingly, we observed that GSK3β and p-GSK3β expression was increased and β-catenin was inhibited following transient transfection of NPC cells with FOXO1, while the levels were reversed after treatment with two FOXO1 siRNAs (Fig. 1k). Furthermore, TCF4 and ZEB1, which are downstream signals of the Wnt/β-catenin pathway, were suppressed (Fig. 1l). Altogether, these data indicate that FOXO1 induces GSK3β to reduce β-catenin/TCF4-mediated tumor stemness and EMT signals.

### FOXO1 induces miR-200b through PI3K/AKT/GSK3β/β-catenin/TCF4-stimulation of ZEB1 to reduce tumor stemness

In a previous study, we used miRNA chip technology to examine the differentially expressed miRNAs in FOXO1-overexpressing NPC cells (Supplementary Fig. 2A, GEO Accession Number: GSE78742) and confirmed that miR-200b is a positive modulator of FOXO1 (Supplementary Fig. 2B). The suppressive effects that ectopic FOXO1 has on stemness, migration, and invasion were reversed by miR-200b expression, as shown by sphere-forming, transwell, and Boyden assays (Supplementary Fig. 2C, D). Western blots revealed a reduction in stemness and EMT factors, such as SOX2, N-Ca, and Vimentin, and the increase in E-Ca in FOXO1-expressing NPC cells was reversed after a miR-200b inhibitor was introduced into these cells (Supplementary Fig. 2E).

Analysis performed using the JASPAR (http://jaspar.genereg.net) database predicted that the human miR-200b promoter region contains three putative ZEB1 binding sites. We used two specific ZEB1 siRNAs (Supplementary Fig. 2F, G) and found that ZEB1 suppression greatly increased miR-200b expression. No significant doses or time-dependent responses were observed following siRNA-mediated reduction of ZEB1 levels (Supplementary Fig. 2H, I), implying that ZEB1 is an upstream regulator of miR-200b.

Chromatin immunoprecipitation (ChIP) showed that endogenous ZEB1 bound to the miR-200b promoter in NPC cells (Supplementary Fig. 2J). Dual-luciferase reporter assays suggested that ZEB1 inhibits the transcriptional activity of the miR-200b promoter (Supplementary Fig. 2K). These results demonstrated that ZEB1 specifically binds to the miR-200b promoter. Then, we observed that the binding activity between ZEB1 and miR-200b was reduced when β-catenin expression was suppressed (Supplementary Fig. 2L). TCF4 and ZEB1 levels were downregulated when an siRNA targeting β-catenin was introduced into the NPC cells (Supplementary Fig. 2M). These results indicate that FOXO1 induces miR-200b expression through β-catenin/TCF4-stimulated ZEB1, which reduced tumor stemness, migration, and invasion of NPC.

### FOXO1 interacts with MYH9

**Fig. 2:**
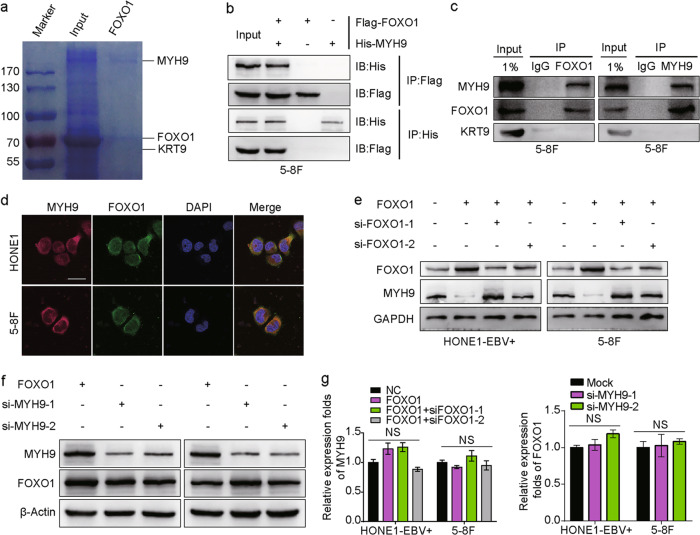
Interaction between FOXO1 and MYH9 in NPC cells. **a** Coomassie brilliant blue staining showed the proteins that interacted with FOXO1 in 5–8F cells and the molecular weights of FOXO1, KRT9 and MYH9. **b** Co-IP experiments detected the interaction of exogenous FOXO1 and MYH9 in 5–8F cells. **c** Co-IP experiments detected the interaction of endogenous FOXO1 and MYH9 in 5–8F cells. **d** Cytosolic colocalization of FOXO1 protein and MYH9 protein in NPC cells is visualized by immunofluorescence staining. Scale bar, 25 μm. **e** MYH9 and FOXO1 expression after FOXO1 was overexpressed or knocked down is shown by western blot analysis. **f** FOXO1 and MYH9 expression after MYH9 was knocked down is shown by western blot analysis. **g** MYH9 mRNA expression is shown after FOXO1 overexpression and knockdown; FOXO1 expression is shown after MYH9 knockdown, and data are normalized to ARF5. One-way ANOVA and Dunnett multiple comparison tests were used for analysis

We used coimmunoprecipitation (Co-IP) and mass spectrometry to explore the relevant binding partners of FOXO1. The analysis yielded several FOXO1-interacting proteins that had high match scores (Supplementary Table 6), including MYH9 (224 kDa band) and Keratin 9 (KRT9, 62 kDa band, Fig. 2a). Exogenous and endogenous Co-IP demonstrated that FOXO1 interacted with MYH9 but not KRT9 in NPC cells (Fig. 2b, c). Additionally, FOXO1 and MYH9 protein colocalization was observed in the cytoplasm of NPC cells using immunofluorescence (Fig. 2d). Altogether, these results suggested that FOXO1 interacts with MYH9 in NPC cells.

Moreover, we observed that MYH9 protein was induced when FOXO1 was suppressed (Fig. 2e), while MYH9 could not regulate FOXO1 expression (Fig. 2f). These data suggest that FOXO1 is upstream of MYH9. Interestingly, using qPCR, we found that MYH9 expression was not suppressed at the mRNA level (Fig. 2g) in FOXO1-overexpressing NPC cells, which suggests a posttranscriptional mechanism for FOXO1-mediated suppression of MYH9.

### MiR-133a-3p directly targets MYH9

**Fig. 3:**
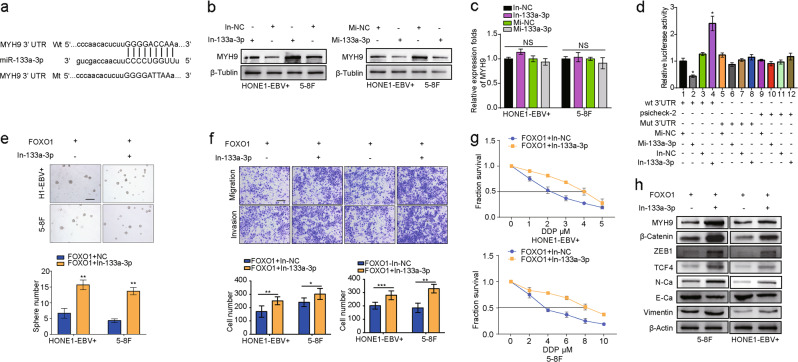
miR-133a-3p directly targets MYH9 to inactivate the Wnt/β-catenin signal. **a** Bioinformatics analysis revealed that the 3’UTR of MYH9 was well matched with the seed sequence of miR-133a-3p. Mutants were generated in the binding region of the MYH9 3’UTR. **b** MYH9 protein levels in miR-133a-3p-overexpressing/suppressing 5–8F cells and HONE1-EBV + cells were detected by western blot analysis. **c** MYH9 mRNA levels in miR-133a-3p-overexpressing/suppressing 5–8F cells and HONE1-EBV + cells were detected by qPCR assays. **d** miR-133a-3p directly targets MYH9, as confirmed by a dual-luciferase reporter assay. One-way ANOVA and Dunnett multiple comparison tests were used for analysis. *P < 0.05. Sphere-forming assay (**e**, scale bar: 500 μm**)**, migration assay, invasion assays (**f**, scale bar: 200 μm**)** and IC50 (**g**) of NPC cells were performed after transfection with a FOXO1 lentiviral vector or miR-133a-3p inhibitor as indicated. Student’s *t*-tests were used for analysis. Mean ± s.d., *P < 0.05; **P < 0.01; ***P < 0.001. **h** Expression of MYH9, β-catenin, ZEB1, TCF4, N-Ca, E-Ca, and Vimentin following miR-133a-3p suppression in FOXO1-overexpressing NPC cells. Abbreviations: Mi-133a-3p: miR-133a-3p mimics; In-133a-3p: miR-133a-3p inhibitor

Bioinformatics analysis revealed that there is a target of the seed sequence of miR-133a-3p in the MYH9 3’UTR (Fig. 3a). Strikingly, western blot analysis showed that miR-133a-3p overexpression downregulated MYH9 in NPC cells, whereas suppression of miR-133a-3p with an inhibitor notably increased MYH9 expression in NPC cells (Fig. 3b). Results from qPCR experiments showed that miR-133a-3p overexpression had little effect on MYH9 transcript levels (Fig. 3c), implying that miR-133a-3p inhibits MYH9 at the protein level. A luciferase activity assay showed that reporter activity from the wild-type MYH9 3’UTR, but not the mutant 3’UTR, was significantly reduced by treatment with miR-133a-3p mimics. Conversely, treatment with miR-133a-3p inhibitors caused robust activation of luciferase activity from the reporter with the wild-type MYH9 3’UTR but not the reporter with the mutant 3’UTR. This effect was not observed in the psiCHECK-2 vector control groups (Fig. 3d). These data demonstrate that MYH9 is a direct target of miR-133a-3p.

To further observe the effect of miR-133a-3p on FOXO1-modulated pathways, the miR-133a-3p inhibitor was transiently transfected into FOXO1-overexpressing NPC cells. Treatment with the miR-133a-3p inhibitor restored cancer stemness, metastatic ability, and DDP chemoresistance (Fig. 3e–g). Furthermore, the levels of N-Ca, Vimentin, MYH9, β-catenin, TCF4, and ZEB1 were notably increased, and E-Ca expression was decreased (Fig. 3h). These results indicate that miR-133a-3p mediates MYH9/GSK3β/β-catenin/TCF4 upstream of FOXO1.

### P53 induces miR-133a-3p by binding to its promoter region

JASPAR (http://jaspar.genereg.net) database analysis predicted that the human miR-133a-3p promoter region contains putative P53-binding sites (from ~−699 to ~−707; Supplementary Fig. S3A). To examine whether P53 regulates miR-133a-3p, we introduced a P53 plasmid into HONE1-EBV + and 5–8F cells (Supplementary Fig. S3B). P53 overexpression greatly increased the expression of miR-133a-3p (Supplementary Fig. S3C). Chromatin immunoprecipitation (ChIP) assays determined that endogenous P53 binds to the miR-133a-3p promoter in NPC cells (Supplementary Fig. S3D). EMSA data further support the idea that P53 specifically binds to the miR-133a-3p promoter (Supplementary Fig. S3E).

Luciferase activities were measured from reporters carrying the wild-type miR-133a-3p promoter during cotransfection with P53, and levels were significantly reduced when the mutant miR-133a-3p promoter vector was cotransfected with P53 (Supplementary Fig. S3F), demonstrating that P53 induced transcriptional activity via the miR-133a-3p promoter. We also found that overexpressing P53 reduced the expression of MYH9, β-catenin, TCF4, and ZEB1 (Supplementary Fig. S3G). Suppression of P53 reversed FOXO1-inactivated stemness (Supplementary Fig. S3H), metastatic activity (Supplementary Fig. S3I), DDP chemoresistance (Supplementary Fig. S3J), and expression of MYH9, β-catenin, TCF4, ZEB1 (Supplementary Fig. S3K), and miR-133a-3p (Supplementary Fig. S3L). These results indicated that P53 induces miR-133a-3p expression by binding to its promoter region.

Further, western blotting assays showed that the expression levels of p-PI3K, p-AKT, c-Myc, and MYH9 were upregulated, while the expression level of P53 was suppressed after knocking down FOXO1 in FOXO1-overexpressing NPC cells (Supplementary Fig. S3M). Furthermore, miR-133a expression was found to be downregulated (Supplementary Fig. S3N). These results were reversed after treating si-FOXO1-treated FOXO1-overexpressing NPC cells with the PI3K-specific inhibitor Ly294002 (Supplementary Fig. S3M, N). ChIP assays showed that the ability of P53 to bind to the miR-133a-3p promoter was further enhanced by Ly294002 treatment in FOXO1-overexpressing NPC cells (Supplementary Fig. S3O). Furthermore, c-Myc was transfected into FOXO1-overexpressing NPC cells, and we observed that the stemness (Supplementary Fig. S3P), migration ability (Supplementary Fig. S3Q), and invasion ability (Supplementary Fig. S3R) of NPC cells were obviously restored. These data indicate that FOXO1 suppresses MYH9 by modulating the PI3K/AKT/c-Myc/P53/miR-133a-3p axis.

### MYH9 interacts with GSK3β and degrades its expression by ubiquitination

**Fig. 4:**
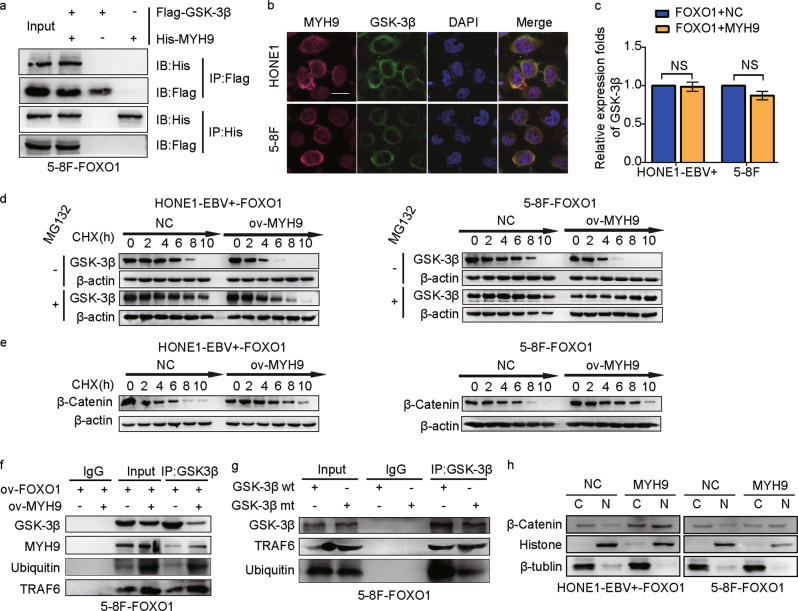
MYH9 interacts with GSK3β and degrades GSK3β by ubiquitination in FOXO1-overexpressing NPC cells. **a** Co-IP detected the interaction of exogenous GSK3β and MYH9 in 5–8F cells. **b** Cytosolic colocalization of GSK3β protein and MYH9 protein in NPC cells was visualized by immunofluorescence staining. Scale bar, 25 μm. **c** GSK3β expression was measured in MYH9-overexpressing NPC cells; one-way ANOVA and Dunnett’s multiple comparison test were used for analysis. **d**, **e** Western blotting and quantification analysis were performed to analyze the effect of MYH9 overexpression on GSK3β and β-catenin stability in NPC cells treated with cycloheximide at different time points and with the presence of MG132. **f** Coimmunoprecipitation analysis of the effect of FOXO1 and MYH9 on the interaction between GSK3β, MYH9, ubiquitin, and TRAF6 in FOXO1-overexpressing NPC cells. **g** Coimmunoprecipitation analysis of GSK3β, TRAF6, and ubiquitin in NPC cells treated with wild-type GSK3β or mutant GSK3β. **h** Nuclear and cytoplasmic extraction assays showed protein levels of β-catenin in the nucleus and cytoplasm of FOXO1-overexpressing NPC cells

Interestingly, using the DOMINE database, which is a protein domain interaction database, we found that the protein kinase domain of GSK3β interacts directly with multiple structures of MYH9. Exogenous Co-IP demonstrated that MYH9 and GSK3β interact in FOXO1-overexpressing 5–8F cells (Fig. 4a). Cytosol colocalization of MYH9 and GSK3β proteins was observed by immunofluorescence in FOXO1-overexpressing HONE1 and 5–8F cells (Fig. 4b). Altogether, these results suggest that MYH9 and GSK3β associate in FOXO1-overexpressing NPC cells.

Surprisingly, we found that the mRNA expression level of GSK3β did not change when a plasmid expressing MYH9 was transfected into FOXO1-overexpressing NPC cells (Fig. 4c). To further investigate the mechanism of GSK3β, 40 μM cycloheximide (CHX) was introduced into FOXO1-overexpressing NPC cells, and we found that MYH9-mediated GSK3β proteins had shorter half-lives than proteins in the control group. Furthermore, we observed that the half-life of GSK3β did not change when cells were treated with a reversible proteasome inhibitor, MG132 (Fig. 4d); however, the half-life of β-catenin was prolonged in MYH9-overexpressing NPC cells compared to the control group (Fig. 4e). Co-IP assays using FOXO1-overexpressing 5–8F cells indicated that MYH9 overexpression facilitated the binding of GSK3β and ubiquitin ligation complex, which contains TRAF6 (Fig. 4f). We further mutated a known ubiquitination site of GSK3β (Lysine 183) and transfected the mutant plasmid into FOXO1-overexpressing NPC cells to observe changes in the binding of GSK3β to ubiquitin. In Fig. 4g, we used a Co-IP assay and observed that the NPC cells transfected with the mutated GSK3β expression plasmid had decreased binding between GSK3β and TRAF6, an ubiquitin-linked enzyme, compared to the control group that was transfected with the wild GSK3β plasmid. These results showed that GSK3β ubiquitination influenced its stability through binding to TRAF6, and lysine 183 was essential for GSK3β poly-ubiquitination in FOXO1-overexpressing NPC cells. In addition, analysis of nuclear and cytoplasmic extracts showed that β-catenin protein levels were increased in the nucleus of MYH9-overexpressing HONE1-EBV + and 5–8F cells. In contrast, the protein level of β-catenin in the cytoplasm was reduced (Fig. 4h). These results demonstrated that MYH9 induced nuclear translocation of β-catenin in NPC cells by regulating ubiquitination of GSK3β to decrease its cellular levels.

### MYH9 reverses FOXO1-mediated inhibition of tumor stemness, metastasis, and DDP chemoresistance in NPC cells

An MYH9 expression plasmid was transfected into FOXO1-overexpressing NPC cells. Cancer stemness, migration, invasion, and DDP chemoresistance were significantly restored, as shown by the sphere-forming (Supplementary Fig. 4A), immunofluorescence (Supplementary Fig. 4B), transwell, and Boyden assays (Supplementary Fig. 4C), and the DDP IC50 (Supplementary Fig. 4D), respectively. MYH9 expression increased N-Ca, Vimentin, β-catenin, and TCF4 protein expression and decreased E-Ca and GSK3β protein expression in FOXO1-overexpressing NPC cells (Supplementary Fig. 4E). These data provide evidence that FOXO1 is upstream of MYH9 in NPC cells.

### GSK3β knockdown reverses the FOXO1-mediated inhibition of tumor stemness, metastasis, and DDP chemoresistance in NPC cells

To explore the role of GKS3β in FOXO1-suppressed stemness, migration, and invasion in NPC cells, we transfected a GSK3β siRNA into FOXO1-overexpressing NPC cells. As shown in the sphere-forming results seen in Supplementary Fig. 5A, NPC cell stemness inhibition was reversed when GSK3β siRNA was introduced into FOXO1-overexpressing NPC cells. Furthermore, migration and invasion were shown to be enhanced in the transwell (Supplementary Fig. 5B) and Boyden assays (Supplementary Fig. 5C), respectively, after GSK3β suppression. GSK3β suppression significantly reversed the FOXO1 sensitizing effects on DDP in NPC cells (Supplementary Fig. 5D). Ectopic treatment with si-GSK3β neutralized FOXO1-modulated upregulation of OCT4, SOX2, N-Ca, Vimentin, and downregulation of E-Ca (Supplementary Fig. 5E). These results indicate that GSK3β suppression can overcome NPC cell stemness, EMT, and sensitizing suppression of DDP by FOXO1.

### CB increases the sensitivity of DDP to FOXO1-overexpressing NPC cells

**Fig. 5:**
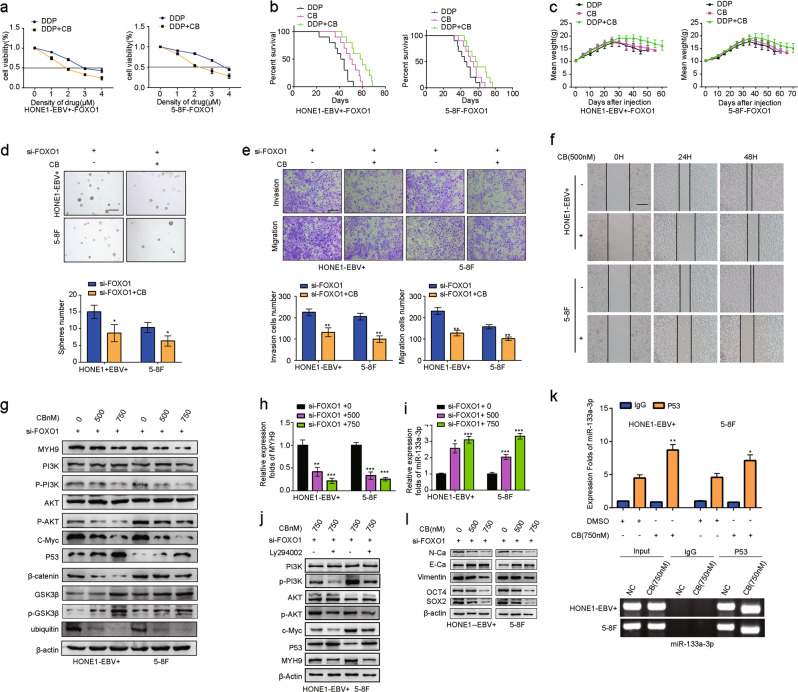
Cinobufotalin increased the sensitivity of FOXO1-overexpressing NPC cells to DDP. **a** Dose–response curves are shown for HONE1-EBV + -FOXO1 and 5–8F-FOXO1 after treatment with DDP and/or CB for 48 h. **b** Survival analysis data show the cumulative overall survival time of mice in the FOXO1 + DDP group, FOXO1 + CB-treated group, FOXO1 + DDP + CB group and control group (*n* = 10, log-rank test). **c** Mean weight of mice in the FOXO1 + DDP group, FOXO1 + CB-treated group, FOXO1 + DDP + CB group and control group (*n* = 10, log-rank test) are shown. Sphere-forming assay (**d**, scale bar: 500 μm), migration assay, invasion assays (**e**, scale bar: 200 μm**)** and scratch assays (**f**, scale bar: 200 μm) of NPC cells were performed after transfection with the FOXO1 lentiviral vector and CB (750 nM) as indicated. Student’s *t*-test. Mean ± s.d. *P < 0.05; **P < 0.01; ***P < 0.001. **g** Expression levels of MYH9, PI3K, p-PI3K, AKT, p-AKT, C-Myc, P53, β-catenin, GSK3β, p-GSK3β, and ubiquitin in FOXO1-suppressed NPC cells treated with CB. **h** MYH9 expression was measured in FOXO1-suppressed NPC cells treated with CB; data are normalized to ARF. One-way ANOVA and Dunnett multiple comparison tests were used for analysis. **P < 0.01 ***P < 0.001. **i** miR-133a-3p expression was measured in FOXO1-suppressed NPC cells treated with CB; data are normalized to U6. One-way ANOVA and Dunnett multiple comparison test. *P < 0.05 ***P < 0.001. **j** Expression levels of PI3K, p-PI3K, AKT, p-AKT, C-Myc, P53, and MYH9 in FOXO1-suppressed NPC cells treated with CB. **k** Amplification of mir-133a-3p-binding sites after Ch-IP using an antibody against P53 in FOXO1-suppressed NPC cells treated with CB. An IgG antibody was used as the negative control. Student’s *t*-test. Mean ± s.d. *P < 0.05; **P < 0.01. **l** Expression of N-Ca, E-Ca and Vimentin, OCT4, SOX2, and β-actin following CB treatment in FOXO1-suppressed NPC cells

Compared with the DDP group, the IC50 value of DDP in the FOXO1-overexpressing NPC cells was significantly reduced when combined with CB (Fig. 5a). Next, we observed that the survival time of nude mice treated with DDP was significantly lower than that of mice treated with the same dose of CB. More interestingly, nude mice with the combined DDP and CB dose showed a longer survival time than the other two groups (Fig. 5b). In addition, the weights of the mice in each group were measured, and the changes in weight are shown in Fig. 5c. We found that the weight of the mice in the DDP group was markedly reduced compared with that in the DDP + CB and CB groups.

### CB inhibits EMT and tumor stemness signals by suppressing MYH9 in FOXO1-suppressed NPC

Using sphere-forming, transwell, Boyden and wound healing assays, we found that CB markedly blocked the formation of tumorspheres, the invasion and the migration induced by treatment of NPC cells with FOXO1 siRNA (Fig. 5d–f). Furthermore, we observed that MYH9 expression was notably reduced at the mRNA and protein levels when CB was introduced into these NPC cells (Fig. 5g, h). Subsequently, we showed that the expression levels of p-PI3K, p-AKT, and c-Myc were suppressed, while that of P53 and miR-133a-3p was induced with CB treatment of FOXO1-suppressed NPC cells (Fig. 5g, i). The expression levels of these factors were markedly reversed when the PI3K inhibitor Ly294002 was added to FOXO1-suppressed NPC cells in addition to CB treatment (Fig. 5j). ChIP assays showed that the binding activity of P53 to the miR-133a-3p promoter was markedly enhanced with CB treatment in FOXO1-suppressed NPC cells (Fig. 5k). Furthermore, the MYH9/GSK3β/β-catenin signal and its downstream tumor stemness and EMT effects were observed to be significantly attenuated in FOXO1-suppressed NPC cells treated with CB, which was accompanied by reduced N-Ca, Vimentin, OCT4, SOX2, and β-catenin protein levels and elevated E-Ca, GSK3β, and p-GSK3β protein levels (Fig. 5g, l). Taken together, these data demonstrated that CB reduced MYH9 via regulating PI3K/AKT/Myc/P53/miR-133a-3p, which then increased GSK3β expression to antagonize β-catenin-activated tumor stemness and EMT signals.

### Clinicopathologic characteristics of FOXO1 and MYH9 expression in NPC cells

**Fig. 6:**
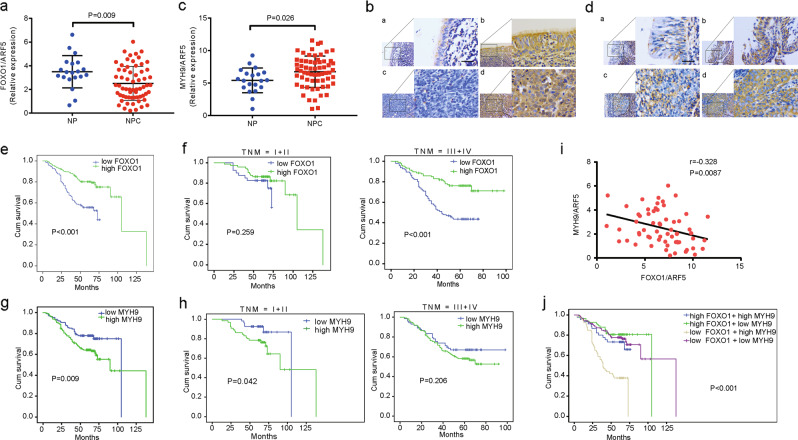
Pathoclinical features of FOXO1 and MYH9 expression and their correlation. **a** Levels of FOXO1 were detected by qPCR in NP and NPC tissue specimens; data are normalized to the ARF5. Student’s *t*-tests were used for analysis, mean ± s.d., *P* = 0.009. **b** IHC staining of FOXO1 in NPC tissues and NP tissues; a: low expression of FOXO1 in NP tissues; b: strong staining of FOXO1 in NP tissues; c: negative expression of FOXO1 in NPC samples; d: strong staining of FOXO1 in NPC samples. Scale bar, 40 μm. **c** Levels of MYH9 were detected by qPCR in NPC and NP tissue specimens; data are normalized to ARF5. Student’s *t*-tests were used for analysis, mean ± s.d., *P* = 0.026. **d** IHC staining of MYH9 in NPC tissues and NP tissues; a: low expression of MYH9 in NP tissues; b: strong staining of MYH9 in NP tissues; c: negative expression of MYH9 in NPC samples; d: strong staining of MYH9 in NPC samples. Scale bar, 40 μm. **e** Kaplan–Meier survival analysis of the overall survival of 321 NPC patients on the basis of FOXO1 expression. A log-rank test was used to calculate *P-*values, *P* < 0.001. **f** A stratified analysis was calculated for the overall survival of 321 NPC patients based on FOXO1 expression levels. A log-rank test was used to calculate *P-*values; *P* = 0.259; *P* < 0.001. **g** Kaplan–Meier survival analysis is shown for the overall survival of 321 NPC patients on the basis of MYH9 expression. A log-rank test was used to calculate *P-*values, *P* = 0.009. **h** A stratified analysis was calculated for the overall survival of 321 NPC patients based on MYH9 expression levels. A log-rank test was used to calculate *P*-values, *P* = 0.042; *P* = 0.206. **i** Correlations between FOXO1 and MYH9 expression levels were calculated. Two-tailed Spearman’s correlation analysis was performed. Mean ± s.d. *P* = 0.0087; *r* = –0.328. **j** Kaplan–Meier survival analysis of the overall survival of 321 NPC patients on the basis of FOXO1 and MYH9 expression. A log-rank test was used to calculate *P-*values, *P* < 0.001

We further confirmed FOXO1 expression is reduced in NPC tissues compared with NP tissues (Fig. 6a, b and Table 1). The clinical characteristics of the NPC patients are summarized in Supplementary Table 8. No significant associations were found between FOXO1 expression and patient age, gender, smoking history, and family history of NPC cancer. However, we observed that FOXO1 expression was negatively correlated with clinical stage (I–II vs. III–IV) (*P* = 0.043), T classification (T1–T2 vs. T3–T4) (*P* = 0.037), *N* classification (N0–N1 vs. N2–N3) (*P* = 0.001), and distant metastasis stage (M0 vs. M1) (*P* = 0.011) (Supplementary Table 8). In addition, we found that NPC patients with high FOXO1 expression had longer survival times than patients with low FOXO1 expression (log-rank test, *P* < 0.001, Fig. 6e). Stratified analysis showed that FOXO1 expression positively correlated with survival times in stages III–IV (P < 0.001) but not in stages I–II (*P* = 0.259, Fig. 6f).

**Table 1:** The expression of FOXO1 in NPC and NP tissues

Group	Case (*n*)	FOXO1 expression		*P-*value*
		Low	High	
Cancer	321	184 (57.3%)	137 (42.7%)	0.009
Normal epithelium	35	12 (34.3%)	23 (65.7%)	

*NPC* nasopharyngeal carcinoma, *NP* normal epithelium

*χ^2^-test was applied to access the expression of FOXO1 in NPC and NP

In contrast to FOXO1 expression, MYH9 expression was significantly increased in NPC tissues compared with normal NP tissues, which was determined by qPCR analysis (Fig. 6c). MYH9 expression was higher in NPC tissues than it was in NP tissues (Fig. 6d and Table 2). As shown in Supplementary Table 9, no significant associations between MYH9 expression and patient age, gender, M classification, smoking history, and family history of NPC cancer were found. However, we observed that MYH9 expression was positively correlated with clinical stages (I–II vs. III–IV) (*P* = 0.026), T classifications (T1–T2 vs. T3–T4) (*P* = 0.007), and *N* classifications (N0–N1 vs. N2–N3) (*P* = 0.031) (Supplementary Table 9). MYH9 expression was negatively correlated with NPC patient survival times (log-rank test, *P* = 0.009, Fig. 6g). Stratified analysis indicated that MYH9 expression was negatively correlated with survival times in stages I–II (*P* < 0.042) but not in stages III–IV (*P* = 0.206, Fig. 6h).

**Table 2:** The expression of MYH9 in NPC and NP tissues

Group	Case (*n*)	MYH9 expression		*P*-value*
		Low	High	
Cancer	321	106 (33.0%)	215 (67.0%)	0.005
Normal epithelium	35	20 (57.1%)	15 (42.9%)	

*NPC* nasopharyngeal carcinoma, *NP* normal epithelium

*χ^2^-test was applied to access the expression of MYH9 in NPC and NP

Furthermore, we analyzed the correlation of FOXO1 and MYH9 mRNA and protein expression and found those levels were negatively correlated with MYH9 mRNA (Fig. 6i; Pearson correlation coefficient, *P* = 0.0087) and protein expression (Supplementary Table 7) (*P* < 0.001). Low FOXO1 expression and high MYH9 expression was associated with a worse prognosis for survival compared to the other expression possibilities for those two factors (high FOXO1 + high MYH9, high FOXO1 + low MYH9, low FOXO1 + low MYH9, log-rank test, *P* < 0.001, Fig. 6j). Univariate and multivariate Cox regression analysis in 321 NPC patients showed that the T classifications, N classifications, M classifications, and FOXO1 expression were independent of prognostic factors (Supplementary Table 10).

## Discussion

Here, we found that FOXO1 significantly inhibited NPC tumor stemness, migration, invasion, and metastasis in vitro and in vivo. Furthermore, we observed that NPC cells overexpressing FOXO1 showed a decreased tumorigenic ability in extreme limiting dilution analyses. These data are consistent with reports in gastric cancer and pancreatic ductal adenocarcinoma but are in contrast to results in glioblastoma.^9–11^ This discrepancy in the role of FOXO1 in different tumors would most likely be due to the different tissue specificities,^21^ which has been similarly reported for other genes in tumors, such as CTGF,^22^ miR-374a,^23^ and certain members of KRTAP subfamilies.^24^ In addition, NPC cells overexpressing FOXO1 were sensitized to DDP chemotherapy in vitro, and FOXO1 overexpression showed prolonged survival times in tumor-bearing mice undergoing DDP treatment in vivo. These data further supported the role of FOXO1 as a significant tumor suppressor in NPC.

Cancer stem cells (CSCs) and the EMT act as crucial factors for the regulation of tumor metastasis and chemoresistance.^6,25^ Determination of the biological properties of FOXO1 allowed us to study its molecular mechanisms. WNT/β-catenin signaling is a crucial for promoting tumor stemness, EMT, and cell cycle progression. We observed that FOXO1 overexpression upregulated GSK3β and downregulated β-catenin. Furthermore, signals downstream of WNT/β-catenin, including the TCF4/ZEB1 signal,^26^ the tumor stemness pathway, and EMT, were shown to be downregulated in FOXO1-overexpressing NPC cells. Inversely, elevated E-Ca and p53 expression was observed in FOXO1-overexpressing cells. Altogether, these results confirmed that FOXO1 suppresses tumor stemness and EMT signals by modulating the GSK3β/β-catenin pathway.

MicroRNAs were found to be key middle-regulators participating in the modulatory network of some tumor-related genes.^27–29^ To investigate the effect of FOXO1 on miRNAs in NPC, we used miRNA chip data and qPCR to screen differential miRNAs and found that miR-200b was significantly upregulated in FOXO1-overexpressing NPC cells. In previous studies, miR-200b had been reported to target BMI-1 and ZEB1 (and others) and suppress tumor stemness and EMT.^30,31^ In this study, we observed that suppressing miR-200b in FOXO1-overexpressing NPC cells restored tumor stemness, migration, and invasion through downregulation of ZEB1. Furthermore, we observed that miR-200b is suppressed by the ZEB1 transcription factor, which is a downstream, positive regulator of β-catenin/TCF4 signaling in NPC cells.^26^ These data demonstrated that FOXO1 induces miR-200b to suppress tumor stemness and EMT through WNT/β-catenin-stimulated TCF4/ZEB1 signals.

FOXO1 has proven to be an important transcription factor.^32^ It is dominantly expressed in the nuclei of many cancers.^33,34^ However, we observed that FOXO1 was predominantly found in the cytoplasm of NPC cells. To better understand its molecular mechanisms, we used Co-IP combined with mass spectrometry and found that MYH9 was a potential binding partner of FOXO1 in NPC cells. Furthermore, we confirmed the interaction of FOXO1 with MYH9 and the colocalization of these factors in the cytoplasm of NPC cells. In addition, we also observed that FOXO1-suppressed MYH9 protein expression but not mRNA expression in NPC cells.

MiRNA-induced suppression of gene expression mainly occurs at the posttranscriptional level.^35–37^ We searched databases (microRNA.org and TargetScan) and found that miR-133a-3p, a tumor suppressor in some tumors,^38,39^ could directly target MYH9. Furthermore, we confirmed that miR-133a-3p, a tumor suppressor, directly downregulated MYH9 protein expression by binding to its 3’UTR in NPC cells. In a subsequent investigation, by searched the JASPAR database, and found p53 as a predicted transcription factor for miR-133a-3p; our experiments showed that direct binding of p53 to the miR-133a-3p promotor induced miR-133a-3p expression in NPC cells.

It is well known that c-Myc, as a classically oncogenic factor,^40^ is a downstream and positive regulator of PI3K/AKT signaling,^41^ but it negatively modulates p53.^41,42^ Our recent study confirmed that there is a PI3K/AKT/c-Myc/P53/miR-133a-3p signaling axis in NPC.^42^ Moreover, our prior study reported that FOXO1-suppressed PI3K/AKT signaling.^12^ Therefore, we speculated that FOXO1-suppressed MYH9 protein expression by regulating the PI3K/AKT/c-Myc/P53/miR-133a-3p pathway. In line with this speculation, we first observed that the cell abilities of cancer stemness, migration and invasion were obviously restored after c-Myc was transfected into FOXO1-overexpressing NPC cells. Furthermore, we found that suppression of PI3K by a specific inhibitor in si-FOXO1-treated FOXO1-overexpressing NPC cells reduced the activation of PI3K/AKT/c-Myc and the activation of MYH9, and it upregulated P53/miR-133a-3p signals. In addition, the binding of P53 to the miR-133a-3p promoter was significantly enhanced in NPC cells. These results indicate that FOXO1-suppressed MYH9 protein expression by regulating the PI3K/AKT/c-Myc/P53/miR-133a-3p pathway

GSK3β is a highly networked kinase that regulates the function of dozens or even hundreds of proteins through binding and/or enzymatic modifications.^43^ Studies have revealed that GSK3β is a crucial factor in the WNT signaling pathway, inhibiting ubiquitination and ultimately affecting tumor stemness and metastatic ability.^44,45^ Therefore, we speculate that MYH9 reversed FOXO1-mediated NPC stemness, migration, and invasion by inducing ubiquitination of GSK3β. In this study, we observed that MYH9 not only interacted with GSK3β but also increased the ubiquitination and degradation of GSK3β protein via the activity of the E3 ubiquitin-linked enzyme TRAF6, which then upregulated β-catenin expression and induced its nuclear translocation in NPC cells. A previous study confirmed that lysine 183 is essential for GSK3β ubiquitination.^46^ Here, we also showed that GSK3β lysine 183 is significant for GSK3β ubiquitination, and it is involved in MYH9-induced GSK3β ubiquitination in NPC cells (data to be shown in another paper).

To prove that FOXO1-suppressed WNT/β-catenin and its downstream signals by downregulating MYH9 protein expression, MYH9 cDNA was transfected into FOXO1-overexpressing NPC cells. We found that overexpressing MYH9 reversed the FOXO1-induced inhibition of NPC tumor stemness, migration, and invasion. Furthermore, MYH9 expression restored the signals suppressed by FOXO1, including downregulation of GSK3β protein; however, it did not recover mRNA expression or stimulation of β-catenin and its downstream tumor stemness and EMT signals. These data suggest that MYH9 is a potential oncogene in NPC, which is inconsistent with a study published in the journal *Science*; that work showed that MYH9 acted as a candidate tumor suppressor in squamous cell carcinoma through regulation of p53 stability.^47^ However, our findings were similar to other studies, which indicated that MYH9 is a candidate oncogene in esophageal squamous cell carcinoma^48^ and breast adenocarcinoma.^49^ Finally, we also determined that GSK3β suppression reversed the FOXO1-induced suppression of NPC tumor stemness, metastatic ability, and DDP chemoresistance via antagonizing β-catenin-activated tumor stemness and EMT signals.

CB, a bufadienolide extracted from toad venom, has been reported to have anticancer effects in lung and liver cancer.^19,20^ However, the comparison of the effect and cytotoxicity between CB and DDP, which is the most common treatment for NPC, has not yet been reported in tumors. Here, we found that the cytotoxicity of the chemical compound CB was significantly stronger than a matched dose of DDP in NPC cells. In addition, we found that CB further reduced the chemoresistance of DDP to FOXO1-overexpressing NPC cells. Noticeably, we subsequently observed that CB treatment not only increased the survival time of nude mice compared to mice treated with the same dosage of DDP but also markedly prolonged the survival time of nude mice when combined with DDP compared to an individual DDP or CB treatment group; these results were generated using in vivo experiments simulating an advanced NPC disease state. These results indicate that CB increases the sensitivity of FOXO1-overexpressing NPC cells to DDP treatment both in vitro and in vivo. Finally, mechanistic analysis confirmed that CB reduced MYH9 levels via downregulation of PI3K/AKT/c-Myc/P53/miR-133a-3p, which increased GSK3β expression to antagonize β-catenin-activated tumor stemness and EMT signals. These data suggest that CB is a more promising anti-tumor agent than DDP for treating NPC. CB combined with DDP is an optional treatment plan for the treatment of NPC with FOXO1-reduced chemoresistance.

Consistent with the roles that FOXO1 and MYH9 play in vitro and in vivo, we observed that FOXO1 expression was significantly decreased, while that of MYH9 was increased in NPC tissues compared with normal NP tissues. FOXO1 expression was negatively correlated with MYH9 expression in NPC tissues. Reduced FOXO1 expression was negatively correlated with clinical progression in NPC patients, but it was positively correlated with NPC survival times. Conversely, elevated MYH9 expression was positively correlated with clinical progression in NPC patients but negatively correlated with NPC survival times. Patients with low FOXO1 and high MYH9 expression had the worst prognoses for survival compared with other groups. These data further demonstrated that reduced FOXO1 and increased MYH9 expression synergistically promoted the development and spread of NPC. Simultaneously, we found through low-stratified analysis that FOXO1 expression was negatively correlated with NPC survival in clinical stages III–IV but not stages I–II, which hinted that FOXO1 loss promoted the middle and late stages of NPC disease. Unlike FOXO1, high MYH9 expression was negatively correlated with survival in NPC patients with clinical stages I–II but not with stages III–IV. In a previous study, MYH9 was reported to be a receptor of the Epstein-Barr virus, allowing entrance of the virus into the nasopharyngeal epithelial cells. MYH9 translocation into the cytoplasm promoted NPC progression^50^ and revealed how MYH9 promoted NPC progression predominantly in clinical stages I–II. Finally, we found that reduced FOXO1 expression was an independent prognostic indicator in NPC patients, which supports the idea that FOXO1 had an important role in the pathogenesis of NPC.

Altogether, our findings elucidate a complex molecular mechanism that involves FOXO1, MYH9, PI3K/AKT/c-Myc, p53, miR-133a-3p, ubiquitin, TRAF6, the GSK3β/Wnt/β-catenin/TCF4/ZEB1 pathway, miR-200b, tumor stemness, and EMT signals in NPC (Supplementary Fig. 6). We demonstrate that FOXO1/MYH9 is a key suppressor of tumor stemness and EMT, thus inducing DPP chemosensitivity in NPC cells. Furthermore, we found that CB is a promising anti-tumor agent that reduced FOXO1-suppressed DDP resistance by antagonizing the FOXO1-interactive protein MYH9 and its-modulated signals in NPC. In summary, our data highlight the critical role of the FOXO1/MYH9 complex in NPC pathogenesis and provide a useful therapeutic option for the treatment of NPC.

## Materials and methods

### In vivo metastasis assays in nude mice

For in vivo metastasis assays, 100 μl (5 × 10^6^) of FOXO1-overexpressing NPC cells or control cells were intravenously injected into the tail vein of each mouse (five mice per group). Lung tissues were then fluorescently imaged using the LT-9MACIMSYSPLUS whole-body imaging system (Lighttools Research, Encinitas, CA, USA). The mice were maintained in a barrier facility on HEPA-filtered racks and fed an autoclaved laboratory rodent diet. All mice were euthanized after 6 weeks of study, and all studies were conducted in accordance with the principles and procedures outlined in the Southern Medical University Guide for the Care and Use of Animals.

### Establishing the subcutaneous xenograft mouse model

The subcutaneous xenograft mouse model was also established to determine tumor formation abilities. Serial dilutions of cells, 1 × 10^6^, 5 × 10^5^, 1 × 10^5^, and 5 × 10^4^, were injected into the mice (*N* = 6 per group), and tumor-initiating frequencies were calculated using extreme limiting dilution analysis (http://bioinf.wehi.edu.au/software/elda/).

### Treatment experiments on nude mice

In vivo experiments were approved by the BEIJING HFK BIOSCIENCE CO., LTD. All mice (BALB/C, nu/nu) were 4-weeks-old, female, and weighed 11–12 g. To establish an NPC mouse model, 6 × 10^5^ FOXO1-overexpressing HONE1 cells or control cells in 0.2 ml buffered saline were intraperitoneally injected into the mice (*N* = 20 each). Tumors were allowed to grow for 3 days, and then the animals were divided into four groups for treatment testing: the FOXO1 + DDP group; the FOXO1 + normal saline (NS) group; the control cell + DDP (Mock) group; and the FOXO1 + NS group. Mice were intraperitoneally injected with NS or DDP every 3 days. For CB treatment in vivo, FOXO1-overexpressing NPC cells were intraperitoneally injected into mice for 10 days, and the mice were divided into three treated groups, and the same dose was given per group (each group = 10): (1) DDP, (2) CB, (3) CB + DDP. The groups of mice were intraperitoneally injected with DDP, CB, or CB + DDP every 3 days, respectively. Changes in mouse body weights and survival times were determined, and survival curves were analyzed using Kaplan–Meier analyses.

### The sphere-forming assay

Cells were dissociated to produce single-cell suspensions and were seeded in six-well ultralow-attachment plates (Corning, Inc., NY, USA) at a density of 5 × 10^3^ cells/well. They were cultured in serum-free medium DMEM/F12 (gibco, NY,USA) with FGF (20 ng/ml, peprotech, Rocky Hill, USA), EGF (20 ng/ml, peprotech, Rocky Hill, USA), and B27 (2%, gibco, NY, USA). Plasmids, siRNAs, mimics, or inhibitors were transfected into the tumorsphere every 3 days. After incubation for 1–2 weeks, tumorspheres were photographed under a microscope and then dissociated into single cells to form new tumorspheres. The size and number of tumorspheres were determined after continuous passaging for three generations.

### The cycloheximide (CHX) chase assay

Cells were transfected with scrambled siRNA control, siRNA, or plasmid and then incubated with 20 μmol/l MG132 (Sigma-Aldrich, MO, USA) for 6 h or were left untreated. Then, at different time points post 50 µg/ml cycloheximide (Abcam, Massachusetts, USA) treatment, cells were harvested and prepared for western blot analysis.

### Tissue specimens

Sixty-three primary fresh NPC samples with tumor node metastasis (TNM) staging and 20 noncancerous fresh nasopharyngeal samples were simultaneously collected from Nanfang Hospital, China, at the time of diagnosis before any therapy. All fresh samples were immediately preserved in liquid nitrogen. Three-hundred and twenty-one paraffin-embedded NPC specimens and 35 NP paraffin-embedded specimens were obtained from Nanfang Hospital, Guangdong Province, China. Consent from the patients and approval from the Ethics Committee of the Nanfang Hospital were obtained before using the clinical samples for research purposes.

### The migration, invasion, and wound healing assays

A transwell (BD Biosciences, NJ, USA) assay was performed to detect cell migration and invasion abilities. Cells were suspended in 100 μl RPMI-1640 without serum and seeded into the top chamber of the transwells that were either coated with Matrigel (BD Biosciences, NJ, USA) or left uncoated, and the bottom chambers were filled with 500 μl RPMI-1640 supplemented with 10% fetal calf serum (FBS). Migrated cells were stained with Giemsa stain (Fig. 1e and Supplementary Figs. S2D and S5B) or crystal violet and then photographed; cells were then quantified by counting the cell numbers in five random fields. Cells were seeded in six-well plates for growth to a confluent monolayer, and scratches were created using a pipette tip. Progression of migration was assessed via photographs taken at initiation and at 0, 24, and 48 h after wounding.

### **Cell culture, statistical analysis, and other materials and methods**

See the online supplementary materials and methods section.

## Supplementary information

supplementary material

## Data Availability

The datasets generated during and/or analyzed during the current study are available from the corresponding author upon reasonable request.
