# Supplementary material for: Chemical compound cinobufotalin potently induces FOXO1-stimulated cisplatin sensitivity by antagonizing its binding partner MYH9
Source: Signal Transduct Target Ther. 2019 Nov 18;4:48. doi: 10.1038/s41392-019-0084-3 (PMC6861228; doi:10.1038/s41392-019-0084-3)
Supplement: Supplementary file 1 — supplementary material [file 41392_2019_84_MOESM1_ESM.docx]

Supplementary Materials for

Chemical compound Cinobufotalin potently induces FOXO1-stimulated cisplatin sensitivity by antagonizing its binding partner MYH9

YongHao Li 1,6, Xiong Liu2,6, Xian Lin3,6, Menyang Zhao1,6, Yanyi Xiao1, Chen Liu1, Zixi Liang1, Zelong Lin1, Renhui Yi1, Zibo Tang1, Jiahao Liu1, Xin Li3, Qingping Jiang4, Libo Li1, Yinyin Xie1, Zhen Liu1,4*, Weiyi Fang1*

1. Cancer Center, Integrated Hospital of Traditional Chinese Medicine, Southern Medical University, Guangzhou 510315, China. 2. Department of Otolaryngology-Head and Neck Surgery, Nanfang Hospital, Southern Medical University, Guangzhou, China. 3. Cancer Institute, Southern Medical University, Guangzhou 510515, China. 4. Key Laboratory of Protein Modification and Degradation, School of Basic Medical Sciences, Affiliated Cancer Hospital and Institute of Guangzhou Medical University, Guangzhou 511436, China. 5. Department of Pathology, Third Affiliated Hospital, Guangzhou Medical University, Guangzhou 510150, China. 6. These authors contribute to the equal work.

Correspondence to: narcissus_jane@163.com; fangweiyi1975@163.com.

**This PDF file includes:**

Materials and Methods

Figures. S1 to S6

Tables. S1 to S10

Materials and Methods

Cell culture

5-8F and HONE1 cells were stored in the Cancer Research Institute of the Southern Medical University (Guangzhou, China). EBV-positive NPC cell line HONE1-EBV+ was kindly provided by Professor S.-W. Tsao, University of Hong Kong. These cells were confirmed to be free of mycoplasma. Both cell lines were cultured in RPMI-1640 (Corning) supplemented with 10% fetal calf serum (Corning) in a humidiﬁed chamber with 5% CO2 at 37°C.

Lentivirus production and infection

Lentiviral particles carrying the pGC-FU-FOXO1-RFP vector and the flanking control sequence (Mock for short) were constructed by GeneChem (Shanghai, China). 5-8F and HONE1-EBV+ cells were infected with the lentiviral vector, and polyclonal cells, with red fluorescent protein signals, were selected for further experiments using fluorescence-activated cell sorting. FOXO1 protein expression (Cat. No. 2880,1:1000, CST) was measured using Western blotting techniques.

Cell transfections

Plasmids were purchased from Vigene Biosciences (Shangdong, China). siRNAs, mimics, and inhibitors were designed and synthesized by Guangzhou RiboBio Co. Ltd (Guangzhou, China) (Supplementary Table 1). The PI3K inhibitor, Ly294002, was purchased from Sigma. Twenty-four hours before transfection, NPC cells were plated onto a cell culture plate or dish (Nest, Biotech, China). Plasmids, siRNAs, mimics, and inhibitors were then transfected into cells using Lipofectamine TM 2000 (Invitrogen Biotechnology Co., Ltd., Shanghai, China) according to the manufacturer’s protocol. Cells were collected after 48–72 h of culture for further experiments.

qPCR

Total RNA was isolated from cells or harvested from tissues. cDNA synthesis was performed using reverse transcription reagents (TaKaRa Bio, Inc., Shiga, Japan), and then cDNA was used as a template for amplification using specific primers (Supplementary Table 2). According to the manufacturer’s instructions, the BIO-RAD T100 and BIO-RAD CFX 96 detection systems were used to perform RT-PCR and qPCR, respectively.

Western blotting

Cell lysates were obtained in lysis buffer, and the protein concentrations were determined using a BCA protein assay kit (Thermo Scientific, Waltham, MA, USA). Proteins were separated by SDS-PAGE and transferred onto polyvinyl difluoride membranes, which were immunoprobed with the corresponding antibodies. The proteins were detected using an enhanced chemiluminescence reagent (Thermo Scientific, Waltham, MA, USA). Antibodies used included anti-FOXO1, MYH9, GSK3β, p-GSK3β, TRAF6, ubiquitin, β-catenin, c-Myc, P53, SOX2, OCT4, E-cadherin, N-cadherin, vimentin, and β-actin. Dilutions and sources of antibodies are shown in Supplementary Table 3. Images were captured using a ChemiDocTM CRS+ Molecular Imager (Bio-Rad, Hercules, CA, USA).

Immunofluorescence and confocal microscopy

Cells were plated on coverslips in a 24-well plate and cultured overnight to allow for cell adherence. After fixation with 4% paraformaldehyde and permeabilization in 0.2% Triton X-100, the cells were incubated with antibodies and then counterstained with 0.2 mg/ml DAPI and visualized using a fluorescence confocal microscope (Carl Zeiss LSM800).

Nuclear and cytoplasmic extraction assay

Cell nuclear and cytoplasmic extraction assay was conducted using NE-PER@ Nuclear and Cytoplasmic Extraction kit (Thermo Scientific Pierce, UK) according to the manufacturer’s instructions. Briefly, cells were harvested and washed with PBS by pipetting, and were then incubated with ice-cold CER I for 10 minutes at 4°C. After incubation, CER II extraction regent was added to the reaction mixture for another 1 minutes and the lysate was centrifuged at 16000 g for 5 minutes. The supernatant (cytoplasmic extract) was carefully transferred into a fresh microcentrifuge tube and stored in ice. The pellet was resuspended in NER extraction regent and incubated for 40 minutes on ice. The suspension was then centrifuged at 16000g for 10 minutes, and the supernatant (nuclear extract) was transferred to a fresh microcentrifuge tube and stored on ice. The proteins were quantitated by BCA protein assay kit, and further analyzed by western blot analysis.

Cinobufotalin(CB)

CB was purchased from MedChem Express (Monmouth Junction, NJ,USA) (HY-N0880). Stock solutions were prepared using dimethyl sulfoxide (DMSO) as a solvent, and further dissolved to make the desired concentrations for experimental use.

Co-immunoprecipitation (Co-IP) assay

Co-IP was carried out using a Pierce Co-Immunoprecipitation kit (Thermo Scientific, Waltham, MA, USA) according to the manufacturer’s instructions. Briefly, total proteins were extracted from cells, and concentrations were quantified. A total of 5mg protein was incubated with 10μg specific antibodies or IgG overnight at 4°C. After elution, the recovered proteins were subjected to silver staining, mass spectrometry, and Western blot analysis. IgG was used as a negative control.

Luciferase reporter assay

Site-directed mutagenesis (mut) of the miR-133a-3p binding site was generated using the GeneTailor Site-Directed Mutagenesis System (Invitrogen, Guangzhou, China). The wt 3’-UTR or mut 3'-UTR were cloned into the psiCHECK-2 vectors to prepare for the luciferase reporter assays. The vector was co-transfected with the miR-133a-3p mimics/inhibitor or the control sequence into cells, and luciferase activity was measured 48 hours after transfection using the Dual-Luciferase Reporter Assay System (Promega Corporation, Madison, WI, USA). A fragment of ZEB1 3’-UTR (wild-type 3’-UTR) was amplified. Site-directed mutagenesis (mut) of the miR-200b binding site was generated using the GeneTailor Site-Directed Mutagenesis System (Invitrogen, Guangzhou, China). The wt 3’-UTR or mut 3'-UTR were cloned into the psiCHECK-2 vectors to prepare for the luciferase reporter assays. The vector was co-transfected with the miR-200b mimics/inhibitor or the control sequence into the cells, and the luciferase activity was measured 48 hours after transfection using the Dual-Luciferase Reporter Assay System (Promega Corporation, Madison, WI, USA).

Chromatin immunoprecipitation (ChIP) assay

Chromatin immunoprecipitation assays were performed using a ChIP assay kit (Thermo Scientific, Waltham, MA, USA). According to the manufacturer’s protocol, chromatin was crosslinked, isolated, and digested with Micrococcal Nuclease to obtain DNA fragments. P53 or IgG was added to the reaction system for immunoprecipitation. After elution and purification, the recovered DNA fragments were subjected to qPCR and/or PCR. IgG served as a negative control.

The electrophoretic mobility shift assay (EMSA)

The electrophoretic mobility shift assay was conducted using an EMSA Kit (BersinBio, Guangzhou, China) according to the manufacturer’s instructions. Nuclear extracts were obtained from cells, and their concentrations were determined using a BCA assay kit. EMSA was performed in a reaction mixture containing nuclear extracts and biotin-labeled probes. Competition or super-shift assays were performed by adding a 100-fold excess of cold competitors (unlabeled wild-type or mutant probes) (Supplementary Table 4), polyclonal rabbit anti-P53 (Cell Signaling Technology) to the reaction mixture. After electrophoresis and incubation, signals were recorded and analyzed.

Immunohistochemistry (IHC) and immunohistochemical staining evaluations

Paraffin sections (2 μm thickness) were prepared from in vivo experiments and used for immunohistochemistry to detect Ki67 (1:100, Abcam), PCNA (1:50, Proteintech), E-cadherin (1:100, Proteintech), N-cadherin (1:100, Proteintech), and Vimentin (1:100, Proteintech) protein expression. The indirect streptavidin-peroxidase method was used based on the manufacturer’s instructions. Stained tissue sections were examined separately by two pathologists.

Statistical analysis

All the data were analyzed using SPSS 23.0 software (SPSS Inc. Chicago, IL, USA). The data are expressed as the means ± SD from at least three independent experiments. Statistical significance was determined using the Student’s two-tailed t-test for two groups, the one-way ANOVA for multiple groups. The skewed data were analyzed using the Wilcoxon rank sum test. Correlation between gene expression and clinicopathologic characteristics were analyzed using the Chi-square test. Log-rank tests were performed on Kaplan-Meier survival curves to elucidate any significant relationships between gene expression and the overall survival of patients. Univariate and multivariate survival analyses were performed using the Cox proportional hazards regression model. All statistical tests were two-sided and a P value of <0.05 was considered statistically significant. * P < 0.05, ** P < 0.01 and *** P < 0.001.


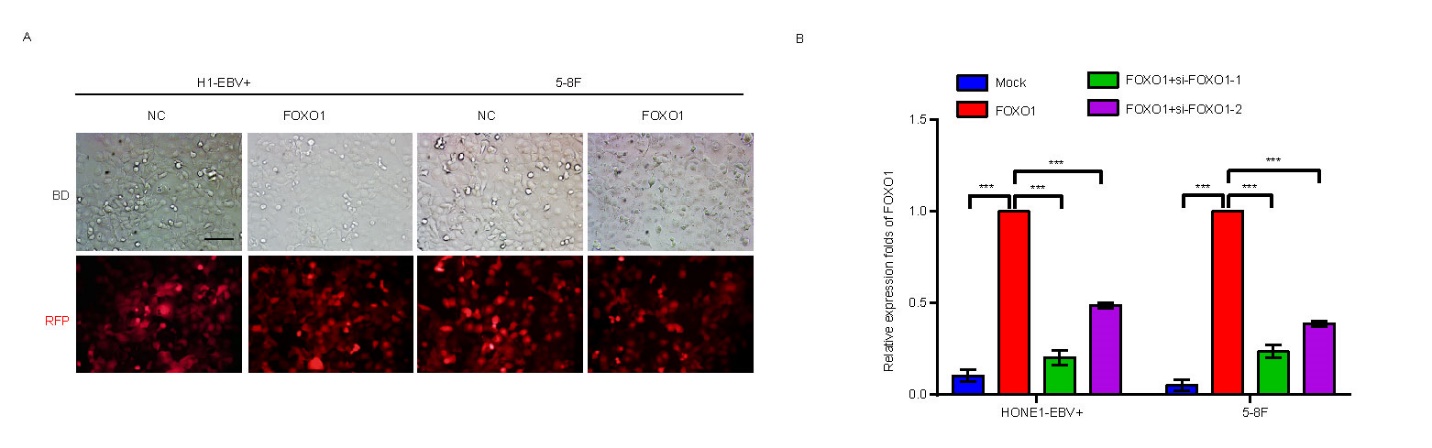


Figure. S1.

FOXO1 attenuates the stemness, migration, invasion and DDP chemoresistance of NPC cells in vitro and in vivo. (A). HONE1-EBV+, 5-8F cells were transfected by lentiviruses containing LV-con or LV-FOXO1, scale bar:50μm. (B). FOXO1 expression was detected after transfection with Mock and/or FOXO1, si-FOXO1.

**
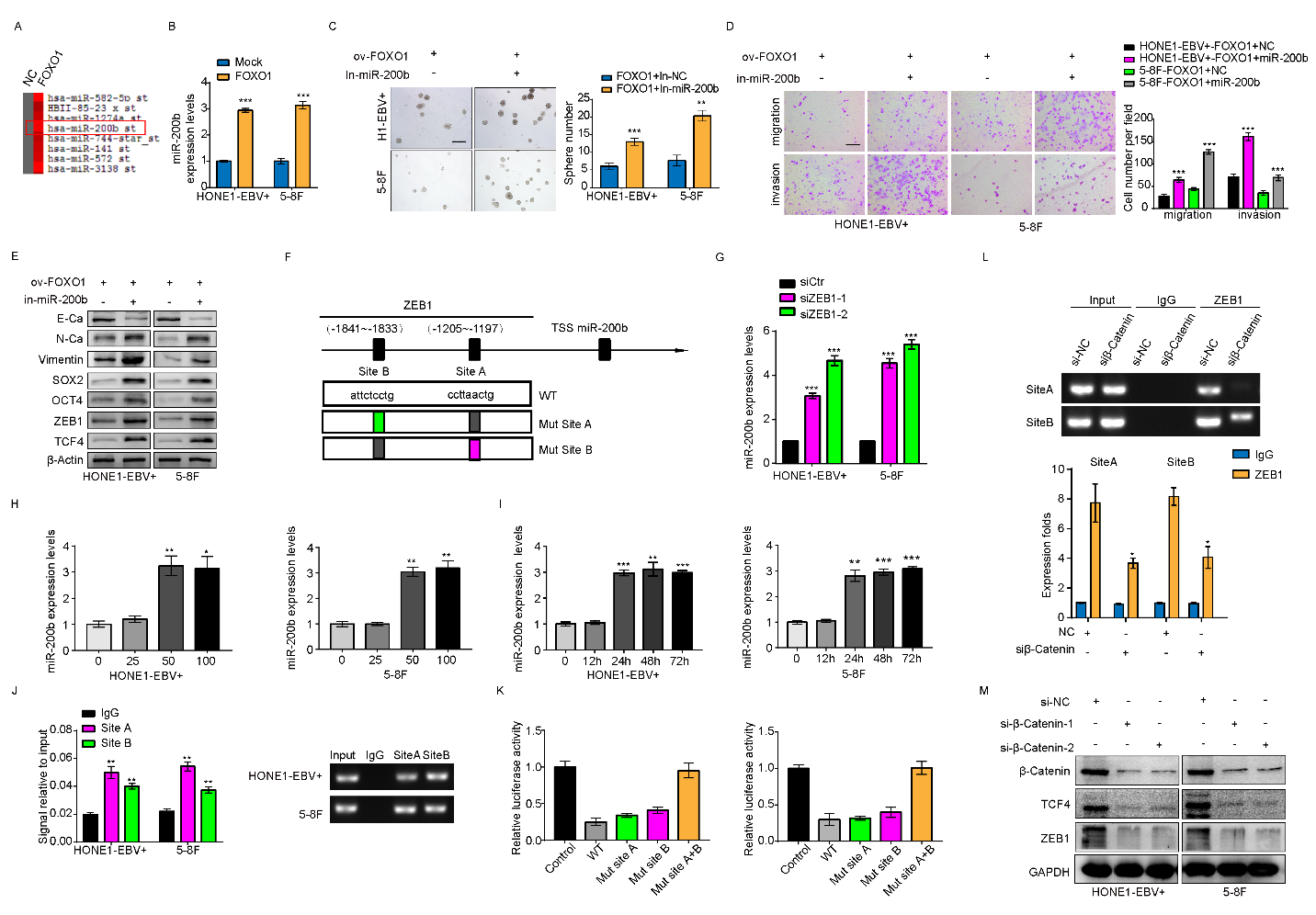
**

Figure. S2.

Endogenous ZEB1 negatively modulates miR-200b expression by directly binding to the promoter region. (A). miRNA expression profiles in FOXO1-overexpressed SUNE1 cells (GEO Accession Number: GSE78742). Red frame, miR-200b. (B).miR-200b expression was measured in FOXO1–overexpressed NPC cells, normalized to U6. Student’s t-test, ***, P < 0.001. Sphere formed assays (C, scale bar, 250μm) and transwell migration and invasion assays (D, scale bar, 100μm) of FOXO1–overexpressing cells treated with miR-200b inhibitor. Student’s t-test,**, P < 0.01， ***，P<0.001. (E). E-Ca, N-Ca, Vimentin, Sox2, OCT4, ZEB1 and TCF4 expression of FOXO1–overexpressing cells treated with miR-200b inhibitor. (F). Schematic diagram of the promoter regions of miR-200b with the putative ZEB1–binding sites (A and B) and the structure of the wild-type (WT) and binding sites mutant (Mut Site A, Mut Site B). (G,H,I) miR-200b expression was measured in ZEB1–suppressed NPC cells, normalized to U6. One-way ANOVA and Dunnett multiple comparison test *, P < 0.05, **，P<0.01; ***，P<0.001. (J). Amplification of ZEB1–binding sites A and B after ChIP using antibody against ZEB1. IgG antibody was used as the negative control, One-way ANOVA and Dunnett multiple comparison test. **，P<0.01. (K). Dual-luciferase reporter assay demonstrated the luciferase activities of the wild type, Mut site A, Mut site B, and Mut site A+B miR-200b promoter in HONE1-EBV+ and 5-8F. One-way ANOVA and Dunnett multiple comparison test. ***, P < 0.001. (L). Amplification of ZEB1–binding sites SiteA and SiteB after ChIP using ZEB1 following β-catenin suppression. *, P < 0.05. (M), β-catenin, TCF4, ZEB1 expression of β-catenin–suppressed cells.


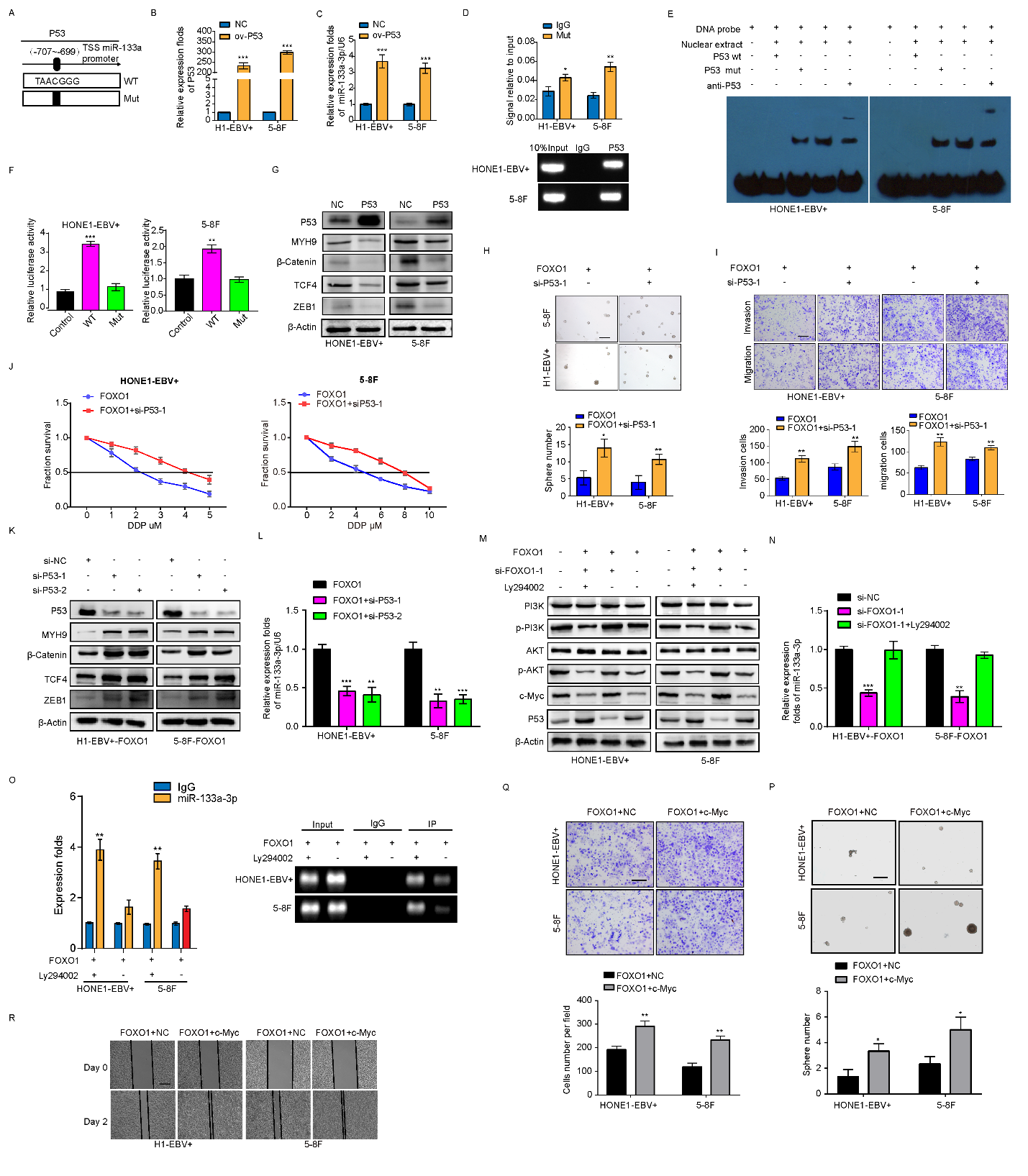


Figure. S3.

Endogenous P53 actively modulates miR-133a-3p expression by directly binding to the promoter region. (A). Schematic diagram of the promoter regions of miR-133a-3p with the putative P53–binding sites and the structure of the wild-type (WT) and binding sites mutant. (B). P53 expression was measured after P53 plasmid was transferred into NPC cells, normalized to ARF. Student’s t-test, ***, P < 0.001. (C). miR-133a-3p expression was measured in P53–overexpressed NPC cells, normalized to U6. Student’s t-test, ***, P < 0.001. (D). Amplification of P53–binding sites after Ch-IP using antibody against P53. IgG antibody was used as the negative control. Student’s t-test, *, P < 0.05; **, P < 0.01. (E). EMSA and supershift assay of P53 binding to miR-133a-3p promoter in HONE1-EBV+ and 5-8F cells. Labeled wild-type probe was incubated without (lane 1) or with (lane 4) cell nuclear proteins in the absence or presence of unlabeled probe (lanes 2–4). Unlabeled wild-type probe (lane 2) and mutant P53 probe (lanes 3 and 4) were used to compete with P53 binding, each at 100-fold excess. Supershift assay (lane 5) was performed using an anti-P53 antibody. (F). Dual-luciferase reporter assay demonstrated the luciferase activities of the wild type, Mut miR-133a-3p promoter in HONE1-EBV+ and 5-8F cells transfected with P53 plasmid. One-way ANOVA and Dunnett multiple comparison test. **P<0.01; ***P<0.001. (G).P53, MYH9, β-catenin, TCF4, ZEB1 expression in NPC. Sphere formation assay (H, scale bar: 250 μm), migration assay, invasion assays (I, scale bar: 100 μm) and IC50(J) of NPC cells were performed after transfection with FOXO1 lentiviral vector or miR-133a-3p inhibitor as indicated. Student’s t-test. Mean±s.d. *, P < 0.05; **P<0.01; ***P<0.001. (K) P53, MYH9, β-catenin, TCF4, ZEB1 expression after P53 suppression. (L) miR-133a-3p expression after P53 suppression. Student’s t-test, Mean±s.d., **P<0.01; ***P<0.001. (M). Expression levels of PI3K, p-PI3K, AKT, p-AKT, c-Myc and P53 treat with FOXO1, si-FOXO1 and Ly294002 in NPC. (N). Expression level of miR-133a-3p with Ly294002 treatment in FOXO1-overexpressing NPC cells. normalized to U6. One-way ANOVA and Dunnett multiple comparison test. **, P < 0.01; ***, P < 0.001. (O). Amplification of P53–binding sites after Ch-IP using antibody against P53 with Ly294002 treatment in FOXO1-overexpressing NPC cells. IgG antibody was used as the negative control. One-way ANOVA and Dunnett multiple comparison test. **, P < 0.01. Sphere formation (P, scale bar: 250 μm), migration (Q, scale bar: 100 μm), and invasion assays (R, scale bar: 100 μm) of FOXO1-overexpressing NPC cells were performed after transfection with c-Myc plasmid as indicated. Student’s t-test，Mean±s.d., *, P < 0.05; **P<0.01; ***P<0.001. Abbreviations:H1-EBV+: HONE1-EBV+


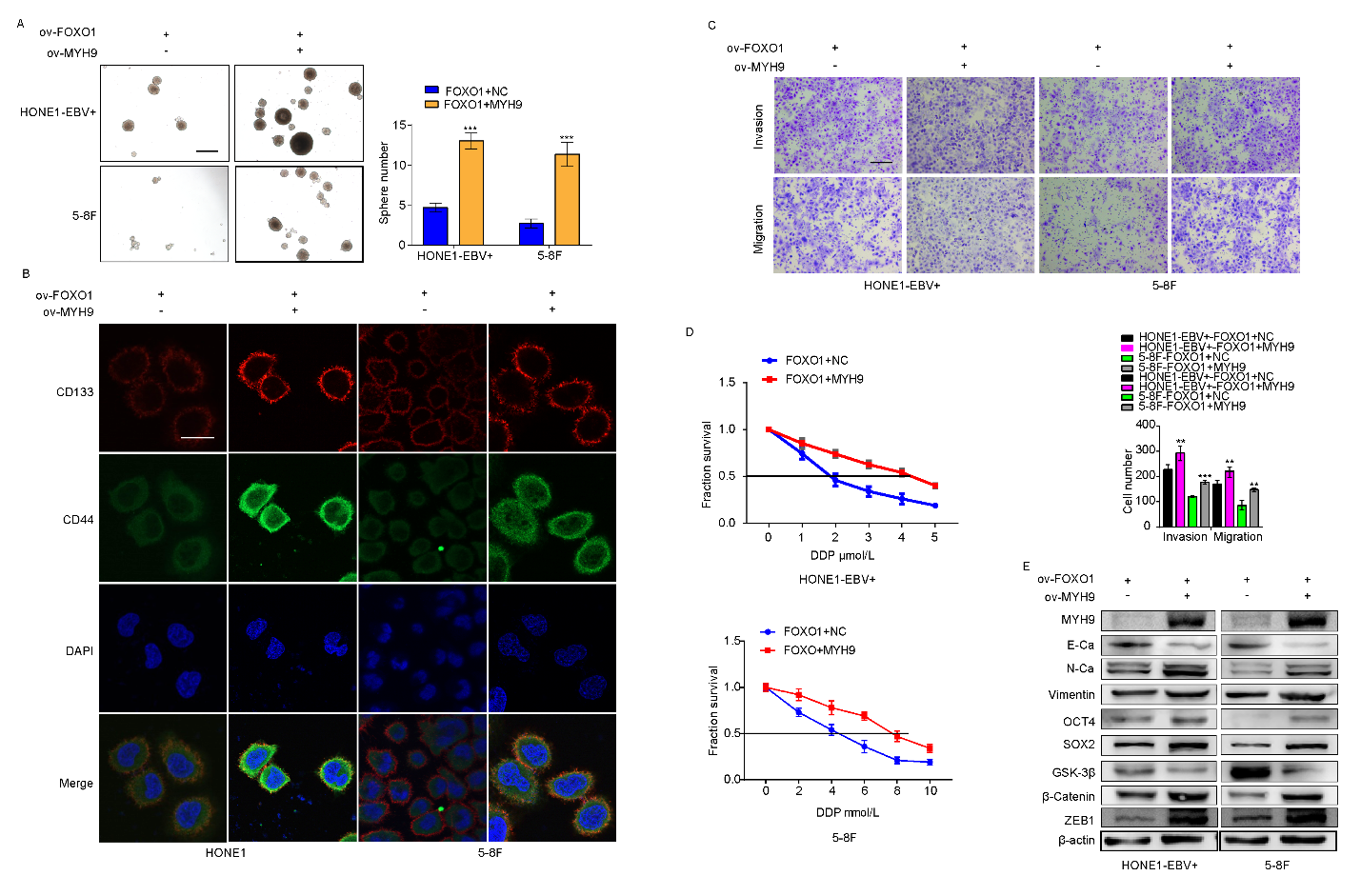


Figure. S4.

Functions of MYH9 in FOXO1-mediated NPC cells. Sphere formed assays (A, scale bar, 250μm), immunofluorescence discrimination assay (B, scale bar:25μm), transwell migration and invasion assays (C, scale bar, 100μm) of FOXO1–overexpressing cells treated with miR-200b inhibitor. Student t test, ***，P<0.001. (D) Dose-response curves of FOXO1-overexpressed NPC cells treated with NC or MYH9 for 48h after treatment with DDP. Parametric generalized linear model with random effects. (E) Expression of MYH9, E-Ca, N-Ca, Vimentin, OCT4, SOX2, GSK-3β, β-catenin, ZEB1 were measured by western blot FOXO1-overexpressed NPC cells treated with NC or MYH9. β-Actin served as a loading control.


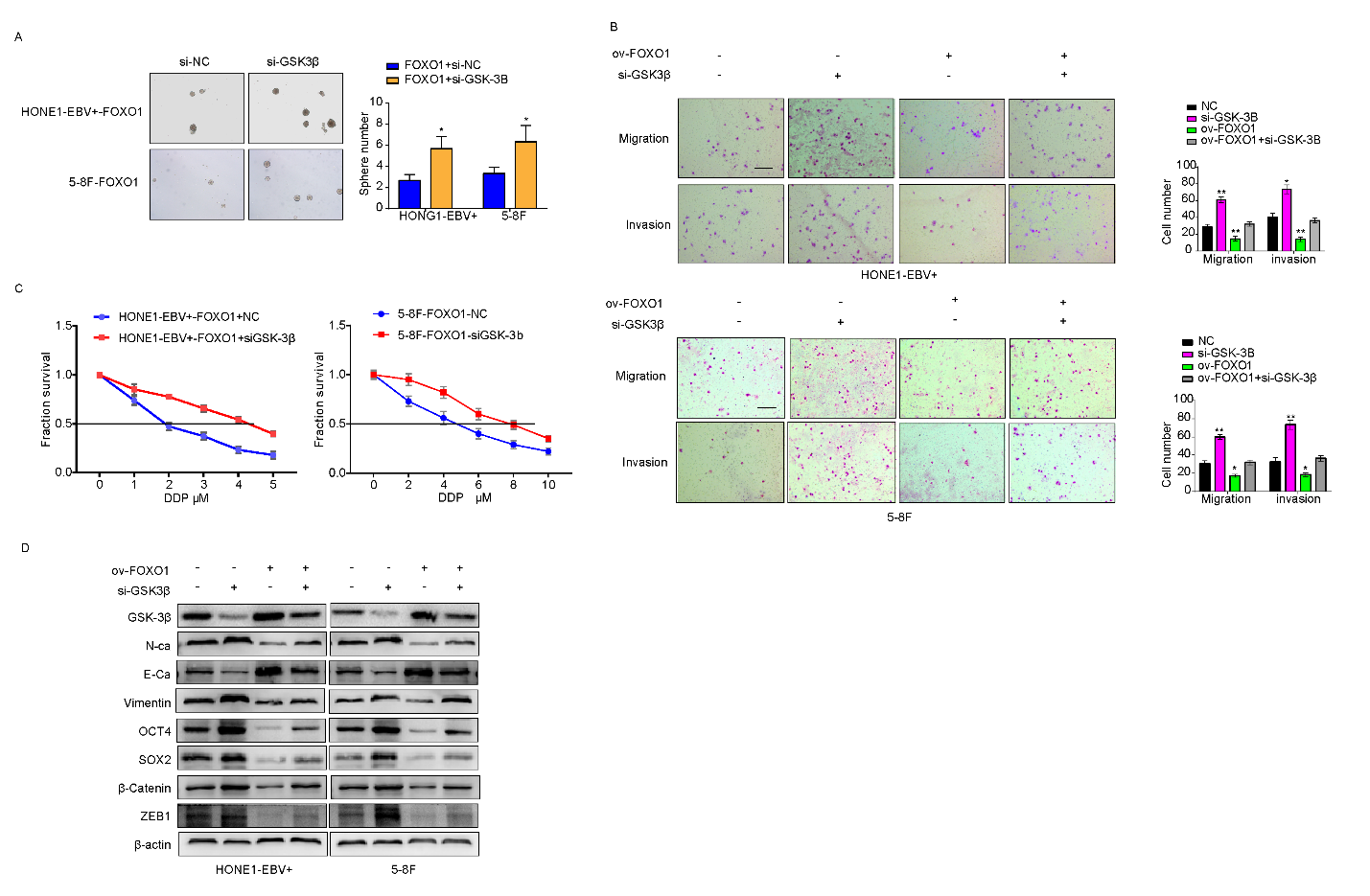


Figure. S5.

GSK-3β reverses FOXO1-medicated inhibitions of tumor stemness, metastasis and DDP chemoresistance in NPC. Sphere formation assay (A, scale bar, 250μm), migraion assay, invasion assays (B, scale bar, 100μm) of NPC cells were performed after transfection with si-GSK-3β in FOXO1-overexpressed NPC cells. Student’s t-test. Mean±s.d., *P<0.05; **P<0.01. (C) Dose-response curves of 5-8F and HONE1-EBV+ treated with si-GSK-3β or FOXO1 48h after treatment with DDP. (D) GSK-3β, N-Ca, E-Ca, Vimentin, OCT4, SOX2, β-catenin, ZEB1 were measured by western blot after transfection with FOXO1 and si-GSK-3β as indicated. β-Actin served as a loading control.


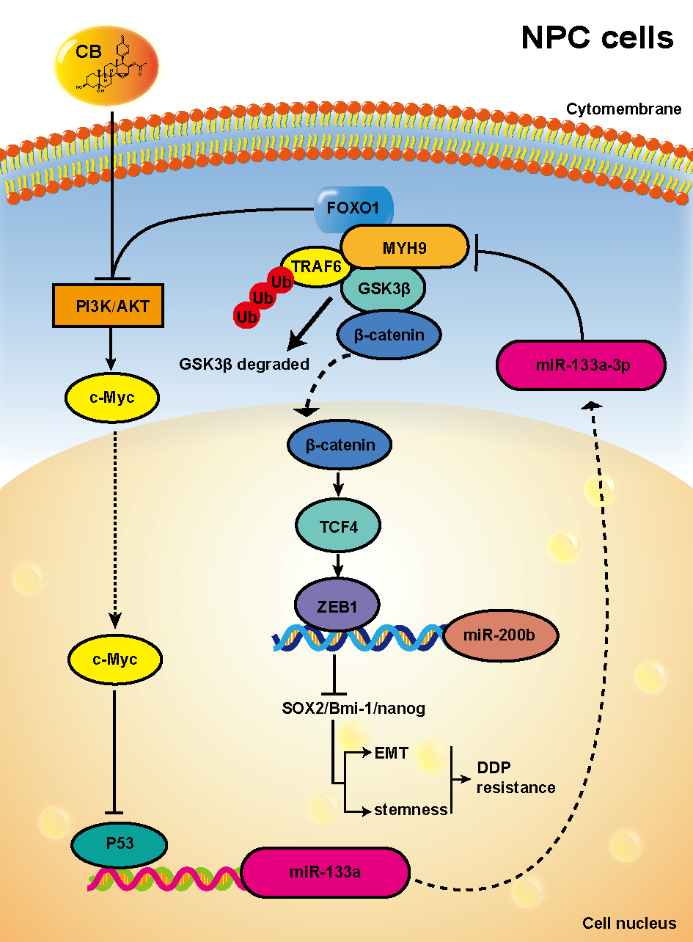


Figure. S6.

Complex molecular mechanism that involves FOXO1, PI3K/AKT/c-Myc, miR-133a-3p, MYH9/GSK-3β/Wnt/β-catenin pathway and miR-200b in NPC

Table S1.

The sequences used in this study.

| Gene | | | | | Sequence |
| --- | --- | --- | --- | --- | --- |
| P53 | 1 | Sense | | 5’GAAAUUUGCGUGUGGAGUA dTdT 3’ | |
|  |  | Antisense | | 3’ dTdT CUUUAAACGCACACCTCAU 5’ | |
|  | 2 | Sense | | 5’CUGCCCUCAACAAGAUGUU dTdT3’ | |
|  |  | Antisense | | 3’ dTdT GACGGGAGUUGUUCUACAA 5’ | |
| FOXO1 | 1 | Sense | | 5’CUGCAUCCAUGGACAACAA dTdT 3’ | |
|  |  | Antisense | | 3’ dTdTGACGUAGGUACCUGUUGUU 5’ | |
|  | 2 | Sense | | 5’ CCAGAUGCCUAUACAAACA dTdT 3’ | |
|  |  | Antisense | | 3’ dTdT GGUCUACGGAUAUGUUUGU 5’ | |
| MYH9 | 1 | Sense | | 5’ GCAAGCUGCCGAUAAGUAU dTdT 3’ | |
|  |  | Antisense | | 3’ dTdT CGUUCGACGGCUAUUCAUA 5’ | |
|  | 2 | Sense | | 5’ GCAAAUUCAUUCGCAUCAA dTdT 3’ | |
|  |  | Antisense | | 3’ dTdT CGUUUAAGUAAGCGUAGUU 5’ | |
| β-catenin | 1 | Sense | | 5’ GAUGGUGUCUGCUAUUGUA dTdT 3’ | |
|  |  | Antisense | | 3’ dTdT CUACCACAGACGAUAACAU 5’ | |
|  | 2 | Sense | | 5’ GGACAAGGAAGCUGCAGAA dTdT 3’ | |
|  |  | Antisense | | 3’ dTdT CCUGUUCCUUCGACGUCUU 5’ | |
| GSK3 β | 1 | Sense | | 5’ AAGAAUCGAGAGCUCCAGAUC dTdT 3’ | |
|  |  | Antisense | | 3’ dTdT UUCUUAGCUCUCGAGGUCUAG 5’ | |
|  | 2 | Sense | | 5’ AAGUAAUCCACCUCUGGCUAC dTdT 3’ | |
|  |  | Antisense | | 3’ dTdT UUCAUUAGGUGGAGACCGAUG 5’ | |
| Negative control |  | | Sense  Antisense | 5’ UUUGUACUACACAAAAGUACUG 3’ | |
|  |  |  |  | 3’ AAACAUGAUGUGUUUUCAUGAC 5’ | |
| miR-133a-3p mimics |  | | Sense | 5’-UUUGGUCCCCUUCAACCAGCUG-3’ | |
|  |  | | Antisense | 5’ CUGCUGGUUGUUGGGGUCCUUU 3’ | |
| Negative control |  | | Sense | 5’ UUUGUACUACACAAAAGUACUG 3’ | |
|  |  | | Antisense | 5’ CUGUUCUUUUGUGUUGUUCUUU 3’ | |
| miR-133a-3p inhibitor | | | | 5’ CUGUGUCCUUUGGUUUUGGCUG 3’ | |
| Inhibitor negative control | | | | 5’ CUGUUCUUUUGUGUUGUUCUUU 3’ | |
| miR-200b mimics |  | | Sense | 5’ UAAUACUGCCUGGUAAUGAUGA 3’ | |
|  |  | | Antisense | 3’ AUUAUGACGGACCAUUACUACU 5’ | |
| miR-200b inhibitor | | | | 5’ UCAUCAUUACCAGGCAGUAUUA 3’ | |
| Inhibitor negative control | | | | 5’ CAGUACUAUUGUCUCGUUCAUA 3’ | |

Table S2.

The primers used in this study.

| Primers name | | Sequence (5’-3’ ) |
| --- | --- | --- |
| FOXO1 | Forward | ACGAGTGGATGGTCAAGAGC |
|  | Reverse | AATTGAATTCTTCCAGCCCGC |
| MYH9 | Forward | AGTTTGTCTCGGAGCTGTGG |
|  | Reverse | GGTTCGTGTTCCTCAGCGTA |
| GSK3 β | Forward | GTCCGATTGCGTTATTTC |
|  | Reverse | AAGAGGTTCTGCGGTTTA |
| β-catenin | Forward | AACTTGCCACACGTGCAATC |
|  | Reverse | TCCACTGGTGAACCAAGCAT |
| β-actin | Forward | ACAGAGCCTCGCCTTTGCC |
|  | Reverse | GATATCATCATCCATGGTGAGCTGG |
| U6 | Forward | CTCGCTTCGGCAGCACA |
|  | Reverse | AACGCTTCACGAATTTGCGT |

Table S3.

A list of antibodies used for WB, ChIP, EMSA, Co-IP, IF and IHC.

| Antibodies name | Cat. No | Company | Species | Dulution |
| --- | --- | --- | --- | --- |
| Flag | F7425 | Sigma | Rabbit | 1:1000 (WB); 1:20 (Co-IP) |
| Ki67 | ab16667 | Abcam | Rabbit | 1:100 (IHC) |
| KRT9 | ab171966 | Abcam | Rabbit | 1:1000 (WB) |
| β-catenin | 8480 | CST | Rabbit | 1:1000 (WB) |
| CD44 | 3570 | CST | Mouse | 1:1000 (WB) ; 1:100 (IF) |
| FOXO1 | 2880 | CST | Rabbit | 1:1000 (WB); 1:20 (Co-IP) ;1:100 (IF) |
| Ubiquitin | 3933 | CST | Rabbit | 1:1000 (WB) |
| MYH9 | 11128-1-AP | Proteintech | Rabbit | 1:1000 (WB); 1:10 (Co-IP); 1:100 (IF); 1:100 (IHC) |
| MYH9 | 60233-1-Ig | Proteintech | Mouse | 1:1000 (WB); 1:100 (IF) |
| GSK3β | 22104-1-AP | Proteintech | Rabbit | 1:1000 (WB); 1:20 (Co-IP); 1:100 (IF); 1:100 (IHC) |
| p-GSK3β | 5558 | CST | Rabbit | 1:1000 (WB) |
| TRAF6 | 66498-1-Ig | Proteintech | Mouse | 1:1000 (WB) |
| c-Myc | 10828-1-AP | Proteintech | Rabbit | 1:1000 (WB) |
| HA | 66006-1-Ig | Proteintech | Rabbit | 1:1000 (WB) ;1:20 (Co-IP) |
| His | 66005-1-Ig | Proteintech | Mouse | 1:1000 (WB); 1:20 (Co-IP) |
| TCF4 | 22337-1-AP | Proteintech | Rabbit | 1:1000 (WB) |
| ZEB1 | 21544-1-AP | Proteintech | Rabbit | 1:1000 (WB) |
| P53 | 10442-1-AP | Proteintech | Rabbit | 1:1000 (WB) |
| AKT | 10176-2-AP | Proteintech | Rabbit | 1:1000 (WB) |
| p-AKT | 66444-1-Ig | Proteintech | Mouse | 1:1000 (WB) |
| PI3K | 60225-1-Ig | Proteintech | Mouse | 1:1000 (WB) |
| SOX2 | 20118-1-AP | Proteintech | Rabbit | 1:1000 (WB) |
| OCT4 | 11263-1-AP | Proteintech | Rabbit | 1:1000 (WB) |
| E-cadherin | 60335-1-Ig | Proteintech | Mouse | 1:1000 (WB); 1:100 (IHC) |
| N-cadherin | 66219-1-Ig | Proteintech | Mouse | 1:1000 (WB); 1:100 (IHC) |
| Vimentin | 10366-1-AP | Proteintech | Rabbit | 1:1000 (WB); 1:1000 (IHC) |
| CD133 | 18470-1-AP | Proteintech | Rabbit | 1:1000 (WB) ; 1:100 (IF) |
| β-actin | 60008-1-Ig | Proteintech | Mouse | 1:5000 (WB) |

Table S4.

The sequences used in Electrophoretic mobility shift assay.

| Gene | Type | | Sequence(5’-3’) |
| --- | --- | --- | --- |
| P53 | probes | wild type | CCCACCTGTGCAGCACCTGCACTCCCATGTGCT |
|  | competitors | wild type | CCCACCTGTGCAGCACCTGCACTCCCATGTGCT |
|  |  | mutant | CCACGGACAGCAGGTGGACGACTCGGTACACCT |

Table S5.

JASPAR database predict ZEB1 binding site.

| Matrix ID | Name | Score | Relative score | Start | End | Strand |
| --- | --- | --- | --- | --- | --- | --- |
| MA0103.2 | ZEB1 | 4.0983 | 0.81498694 | 1197 | 1205 | + |
| MA0103.2 | ZEB1 | 3.96029 | 0.81264374 | 1833 | 1841 | + |
| MA0103.2 | ZEB1 | 3.34317 | 0.80216644 | 1402 | 1410 | + |

Table S6.

Interacting proteins of FOXO1 identified by Co-IP/MS analysis

| Accession | Score | Mass | Protein description |
| --- | --- | --- | --- |
| MYH9_HUMAN | 6425 | 227646 | Myosin-9 OS=Homo sapiens GN=MYH9 PE=1 SV=4 |
| K1C9_HUMAN | 3027 | 62255 | Keratin, type I cytoskeletal 9 OS=Homo sapiens GN=KRT9 PE=1 SV=3 |

Table S7.

The correlation of MYH9 and FOXO1 in NPC tissues.

| Group | Case *(n)* | MYH9 expression | FOXO1 expression | | *P^*^*  value |
| --- | --- | --- | --- | --- | --- |
|  |  |  | Low | High |  |
| Cancer | 321 | Low | 29(9.0%) | 77(24.0%) | 0.000 |
|  |  | High | 155(48.3%) | 60 (18.7%) |  |

NPC, Nasopharyngeal carcinoma; NP, normal epithelium.

^*^ꭓ^2^ test was applied to access the expression of FOXO1 and MYH9 in NPC and NP.

Table S8.

Correlation between the clinicopathologic characteristics and expression of FOXO1 in NPC.

| Characteristics | n | FOXO1 expression | | P^*^value |
| --- | --- | --- | --- | --- |
|  |  | Low | High |  |
| Age(years) |  |  |  |  |
| <50 | 165 | 68(41.2%) | 97(58.8%) | .508 |
| ≥50 | 156 | 70(44.9%) | 86(55.1%) |  |
|  |  |  |  |  |
| Gender |  |  |  |  |
| Male | 98 | 37(37.8%) | 61(62.2%) | .198 |
| Female | 222 | 101(45.5%) | 121(54.5%) |  |
|  |  |  |  |  |
| Clinical stage |  |  |  |  |
| I-II | 113 | 40(35.4%) | 73(64.6%) | .043 |
| III-IV | 208 | 98(47.1%) | 110(52.9%) |  |
|  |  |  |  |  |
| T classification |  |  |  |  |
| T1-T2 | 220 | 86(39.1%) | 134(60.9%) | .037 |
| T3-T4 | 101 | 52(51.5%) | 49(48.5%) |  |
|  |  |  |  |  |
| N classification |  |  |  |  |
| N0-N1 | 153 | 51(33.3%) | 102(66.7%) | .001 |
| N2-N3 | 168 | 87(51.8%) | 81(48.2%) |  |
|  |  |  |  |  |
| M classification |  |  |  |  |
| M0 | 290 | 118(58.3%) | 172(41.7%) | .011 |
| M1 | 31 | 20(41.8%) | 11(58.2%) |  |
|  |  |  |  |  |
| Smoking history |  |  |  |  |
| No | 249 | 109(43.8%) | 140(56.2%) | .598 |
| Yes | 72 | 29(40.3%) | 43(59.7%) |  |
|  |  |  |  |  |
| Family history of cancer |  |  |  |  |
| No | 302 | 132(43.7%) | 170(56.3%) | .300 |
| Yes | 19 | 6(31.6%) | 13(68.4%) |  |

Table S9.

Correlation between the clinicopathologic characteristics and expression of MYH9 in NPC.

| Characteristics | n | MYH9 expression | | P^*^value |
| --- | --- | --- | --- | --- |
|  |  | Low | High |  |
| Age(years) |  |  |  |  |
| <50 | 165 | 72(43.6%) | 93(56.4%) | .125 |
| ≥50 | 156 | 55(35.3%) | 101(64.7%) |  |
|  |  |  |  |  |
| Gender |  |  |  |  |
| Male | 99 | 41(41.8%) | 58(58.2%) | .651 |
| Female | 222 | 86(38.7%) | 136(61.3%) |  |
|  |  |  |  |  |
| Clinical stage |  |  |  |  |
| I-II | 113 | 54(47.8%) | 59(52.2%) | .026 |
| III-IV | 208 | 73(35.1%) | 135(64.9%) |  |
|  |  |  |  |  |
| T classification |  |  |  |  |
| T1-T2 | 220 | 98(43.6%) | 122(56.4%) | .007 |
| T3-T4 | 101 | 29(29.8%) | 72(70.2%) |  |
|  |  |  |  |  |
| N classification |  |  |  |  |
| N0-N1 | 160 | 70(45.8%) | 83(54.2%) | .031 |
| N2-N3 | 161 | 57(33.9%) | 111(66.1%) |  |
|  |  |  |  |  |
| M classification |  |  |  |  |
| M0 | 290 | 118(39.7%) | 172(60.3%) | .208 |
| M1 | 31 | 9(37.5%) | 22(62.5%) |  |
|  |  |  |  |  |
| Smoking |  |  |  |  |
| No | 249 | 98（39.4%) | 151(60.6%) | .888 |
| Yes | 72 | 29(40.3%) | 43(59.7%) |  |
|  |  |  |  |  |
| Family history of cancer |  |  |  |  |
| No | 302 | 119(39.4%) | 183(60.6%) | .815 |
| Yes | 19 | 8(31.6%) | 11(68.4%) |  |

Table S10.

Univariate and multivariate Cox regression analysis in 321 NPC patients

| **Parameters** | **Univariate analysis** | | | **Multivariate analysis** | | |
| --- | --- | --- | --- | --- | --- | --- |
|  | HR | 95% CI | *p** | HR | 95% CI | *p** |
| **Sex** |  |  |  |  |  |  |
| Male vs. Female | 1.336 | 0.861-2.071 | 0.196 |  |  |  |
| **Age (y)** |  |  |  |  |  |  |
| ＜50 vs. ≥50 | 1.379 | 0.935-2.034 | 0.105 |  |  |  |
| **T classification** |  |  |  |  |  |  |
| T1-T2 vs. T3-T4 | 2.402 | 1.623-3.553 | 0.000 | 2.322 | 1.402-3.845 | 0.001 |
| **N classification** |  |  |  |  |  |  |
| N0-N1 vs. N2-N3 | 2.064 | 1.380-3.086 | 0.000 | 1.795 | 1.006-3.203 | 0.048 |
| **M classification** |  |  |  |  |  |  |
| M0 vs. M1 | 5.503 | 3.256-9.302 | 0.000 | 4.930 | 2.726-8.914 | 0.000 |
| **Tumor stage** |  |  |  |  |  |  |
| Ⅰ-Ⅱ vs. Ⅲ-Ⅳ | 2.703 | 1.668-4.379 | 0.000 | 0.830 | 0.373-1.846 | 0.648 |
| **Smoking** |  |  |  |  |  |  |
| Yes vs. No | 1.208 | 0.774-1.885 | 0.405 |  |  |  |
| **Family history of cancer** |  |  |  |  |  |  |
| Yes vs. No | 0.045 | 0.01-1.333 | 0.073 |  |  |  |
| **FOXO1 expression** |  |  |  |  |  |  |
| Low vs. High | 0.384 | 0.257-0.573 | 0.000 | 0.317 | 0.137-0.734 | 0.007 |
| **MYH9 expression** |  |  |  |  |  |  |
| Low vs. High | 1.749 | 1.144-2.673 | 0.000 | 0.978 | 0.218-4.398 | 0.977 |
| **FOXO1 vs. MYH9**  Low vs. High | 1.024 | 0.861-1.218 | 0.791 | 1.420 | 0.630-3.202 | 0.398 |

*p < 0.05 statistically signiﬁcant.

T, tumor size;

N, lymph node; M, distant metastasis; HR, hazard ratio; 95%CI, 95% confidence interval.
